# *Pleurotus ostreatus* Grown on Agro-Industrial Residues: Studies on Microbial Contamination and Shelf-Life Prediction under Different Packaging Types and Storage Temperatures

**DOI:** 10.3390/foods12030524

**Published:** 2023-01-24

**Authors:** Sami Abou Fayssal, Zeina El Sebaaly, Youssef N. Sassine

**Affiliations:** 1 Department of Agronomy, Faculty of Agronomy, University of Forestry, 10 Kliment Ohridski Blvd, 1797 Sofia, Bulgaria; 2 Department of Plant Production, Faculty of Agriculture, Lebanese University, Beirut 1302, Lebanon

**Keywords:** agro-industrial wastes, circular economy, health risk, modified atmosphere packaging, mushrooms, RSM modeling, shelf-life, total microbial count, vacuum packaging

## Abstract

The short shelf-life of mushrooms, due to water loss and microbial spoilage, is the main constraint for commercialization and consumption. The effect of substrate type combined with different temperatures and packaging conditions on the shelf-life of fresh *Pleurotus ostreatus* is scantily researched. The current study investigated the shelf-life of fresh oyster mushrooms grown on low (0.3, 0.3, 0.17) and high (0.7, 0.7, 0.33) rates of olive pruning residues (OLPR), spent coffee grounds (SCG), and both combined residues (OLPR/SCG) with wheat straw (WS), respectively, at ambient (20 °C) and 4 °C temperatures under no packaging, polyethylene plastic bag packaging (PBP), and polypropylene vacuum bag packaging (VBP). Results showed that at ambient temperature OLPR/SCG mushrooms PBP-bagged had an increased shelf-life by 0.5–1.2 days in comparison with WS ones. The predictive models adopted to optimize mushroom shelf-life at ambient temperature set rates of 0.289 and 0.303 of OLPR and OLPR/SCG, respectively, and PBP as the most suitable conditions (9.18 and 9.14 days, respectively). At 4 °C, OLPR/SCG mushrooms VBP-bagged had a longer shelf-life of 2.6–4.4 days compared to WS ones. Predictive models noted a maximized shelf-life of VBP-bagged mushrooms (26.26 days) when a rate of 0.22 OLPR/SCG is incorporated into the initial substrate. The combination of OLPR and SCG increased the shelf-life of fresh *Pleurotus ostreatus* by decreasing the total microbial count (TMC) while delaying weight loss and veil opening, and maintaining carbohydrate content, good firmness, and considerable protein, in comparison with WS regardless the storage temperature and packaging type.

## 1. Introduction

Mushrooms are horticultural crops that have a short shelf-life affected by internal and external factors. Among the internal ones, high moisture content is the main factor for mushroom deterioration [1]. After harvest, mushroom respiration and transpiration rates and enzymatic activities increase dramatically and are well associated with decreases in nutritional composition and the emergence of microbial populations [2,3]. The lack of protective cuticles on the skin of mushrooms besides storage conditions is the most reported external factor affecting their shelf-life period [4].

*Pleurotus ostreatus* (oyster mushroom) is generally grown on wheat straw (WS) substrate [5,6] and a variety of agro-industrial residues as partial or complete substitutes for WS [7,8,9,10,11]. This mushroom holds several nutritional attributes including fatty and amino acids responsible for tremendous improvements in the human diet and health [12,13]. The choice of adequate substrates and supplements is mainly based on their capability to improve mushroom yield and quality [14,15,16]. This also ultimately improves mushroom shelf-life after their harvest [17]. Although the incorporation of agro-industrial residues in *P. ostreatus* substrate showed improvements in yield and quality of produced mushrooms, the effect of the former on the mushroom’s shelf-life (detected mainly by browning and veil opening) is scantily investigated.

In order to overcome the fast deterioration of mushrooms, several techniques related to storage form, packaging type, and storage temperature were developed in the last decades. Among these techniques, modified atmosphere packaging (MAP) proved a relatively long shelf-life period for mushrooms during storage at low temperatures [18]. The preservation of mushroom quality is a key factor in their commercialization [19,20,21]. In Lebanon, the average price of 1 kg mushrooms in 2022 ranged between USD 1.82 and USD 2.86 [22]. Based on the economic inflation and dramatic depreciation of the Lebanese pound, mushroom prices are considered high nowadays. Therefore, there is a need to find suitable preservation techniques for mushrooms. In this context, MAP showed successfully to overcome the shortness of mushroom shelf-life, which is generally paramount to great economic losses and reduction in quality, thus non-marketability and low consumption [23,24,25].

Main studies are nowadays focused on methods aiming at the maximization of mushroom shelf-life during storage through freezing, drying, canning, and irradiation, in addition to several types of packaging [26], resulting in a significant delay of microbial spoilage. However, these studies do not take into consideration that around half of the world’s mushroom production is consumed in a fresh state [27] and sometimes without any treatment, including refrigeration or cold storage. Cold preservation arises as one of the most cost-effective methods to preserve vegetables [28], including mushrooms [29], as it delays microbial spoilage and preserves to some extent the product’s nutritional quality. The shelf-life of mushrooms is also influenced by the physical state of mushrooms (dried or fresh) and the implementation of additives or preservative substances. It depends on the aim of the consumer: fresh and timely consumption or long-term preservation.

The use of predictive models in the field of mushroom production has been reported recently on other species. For instance, artificial neural networks (ANN) and other models were used to predict the uptake of heavy metals by *Agaricus bisporus* grown on commercial WS and dairy wastewater and the resultant health risk [30,31]. Moreover, the uptake prediction of metal elements by the same species grown on materials containing sewage sludge was successfully assessed using regression models [32]. Kinetic modeling successfully predicted the delignification and heavy metals uptake by *Lentinula edodes* mushrooms grown on dairy and sugar mill wastewaters [33].

This study is the first that aims to evaluate: (a) the effect of separate and combined agro-industrial residues on mushroom shelf-life and microbial contamination; (b) the effect of packaging type and storage temperature on the shelf-life and microbial contamination on these mushrooms; (c) the optimization of storage conditions for a maximized shelf-life period using response surface methodology (RSM); (d) the potential health risks during the storage of mushrooms grown on agro-industrial residues and following the aforementioned storage conditions using the total microbial count (TMC) methodology; and (e) the variation in mushroom physicochemical properties and nutritional quality during storage.

## 2. Materials and Methods

### 2.1. Experimental Materials

**Figure 1:**
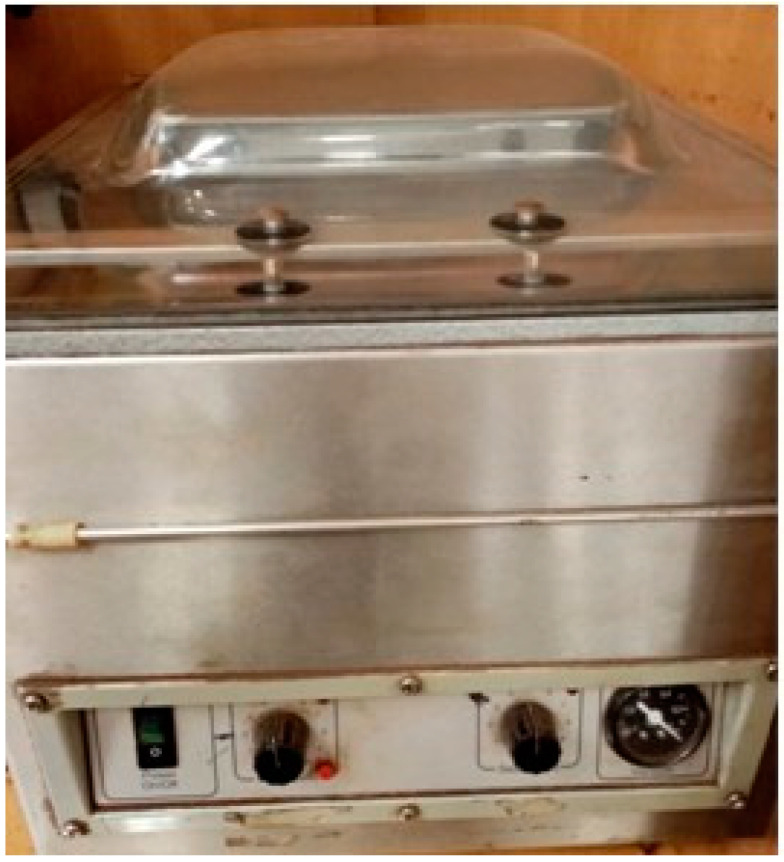
Adopted single-chamber vacuum sealer.

*P. ostreatus* (summer strain oyster) mushrooms were freshly procured from a private mushroom farm (33.772996° N, 35.981931° E) directly after harvest. Harvesting was carried out with high precaution to avoid any mechanical damage. Mushrooms were selected following uniformity in size, shape, and freedom from any defect. They were grown on the following substrates: wheat straw (WS), olive pruning residues (OLPR) in mixtures with WS (*v*/*v*), spent coffee grounds (SCG) in mixtures with WS (*v*/*v*), and both OLPR and SCG in mixtures with WS (*v*/*v*) based on earlier research [5,7,12]. WS substrate (control) had null OLPR, SCG, and OLPR/SCG rates, OLPR mixtures with WS had OLPR rates of 0.3 and 0.7 (0.3:0.7; 0.7:0.3), SCG mixtures with WS had SCG rates (ratios) of 0.3 and 0.7 (0.3:0.7; 0.7:0.3) and OLPR/SCG mixtures with WS had OLPR/SCG rates of 0.17 and 0.33 (0.17:0.17:0.66; 0.33:0.33:0.33). Whole mushrooms (no slicing performed) were used in the current study. Experimental mushrooms were subjected to two storage conditions: ambient temperature (20 °C) and 4 °C, and three packaging conditions: no packaging, plastic (polyethylene) bag packaging, and vacuum (polypropylene) bag packaging, to assess their shelf-life. A 100 mm opening was left in the seal of polyethylene plastic bags (PBP) to reduce the respiration rate of fresh mushrooms without accumulating the produced CO_2_ in the bags’ environment [34]. Vacuum packaging of mushrooms in polypropylene bags (VBP) was performed using a single-chamber vacuum sealer for fruit and vegetables (HK-280, GAMITA PAK-IT PTE. LTD., Singapore) (Figure 1).

The bags were aligned with the seal bar to let the air be drawn out and the latter fused the bag altogether during the process for 30 s, which obtained a tight seal product. The moisture content of fresh mushrooms was measured in triplicates. Briefly, 5 g of mushrooms belonging to each treatment were taken randomly and sliced into small pieces, then crushed and dried for 10 min in a moisture analyzer (M5-Thermo A64M).

### 2.2. Experimental Design

The plan for M_1_, M_2_, and M_3_ shelf-life (mushrooms grown on WS and OLPR mixtures, on WS and SCG mixtures, and WS and OLPR/SCG mixtures, separately) was designed in Design-Expert software (Version 11, StatEase Corp, Minneapolis, MN, USA). A methodology based on central composite design (CCD) was adopted to optimize mushroom shelf-life and model construction. Thirteen triplicates of experimental trials (runs) had a configuration of CCD based on three levels of packaging type (*X*_1_: −1: vacuum bag packaging; 0: no packaging; and +1: plastic bag packaging) and OLPR, SCG, and OLPR/SCG rates (*X*_2_: −1: OLPR, SCG, and OLPR/SCG rates = null; 0: OLPR, SCG, and OLPR/SCG rates = 0.3, 0.3 and 0.17, respectively; +1: SCG, OLPR and SCG/OLPR rates = 0.7, 0.7 and 0.33, respectively) were performed. The design and setup of experimental variables for mushroom shelf-life are given in Table 1.

By using the prepared CCD matrix, the shelf-life of M_1_ (*Y*_1_: days), shelf-life of M_2_ (*Y*_2_: days), and shelf-life of M_3_ (*Y*_3_: days) were optimized using a second-order polynomial quadratic model following the studies of Kumar et al. [35,36]. The adopted model followed the form given in Equation (1). It showed that the variance for each factor was partitioned into linear, quadratic, and interactive components [37].

(1) $$Y=\beta_{0}+\overset{n}{\underset{i=1}{\sum }}\beta_{i}X_{i}+\overset{n}{\underset{i=1}{\sum }}\beta_{ii}X_{i}^{2}+\overset{n-1}{\underset{i=1}{\sum }}\overset{n}{\underset{j=i+1}{\sum }}\beta_{ij}X_{i}X_{j}$$

Herein, *β*_0_ is the main model coefficient (constant), *β_i_* is the coefficient for individual input factor, *β_ii_* is the quadratic coefficient, and *β_ij_* is the interactive coefficient for the input model terms, i.e., *X_i_* and *X_j_* refer to the levels of independent variables (packaging type and OLPR, SCG, OLPR/SCG rates, respectively).

### 2.3. Total Microbial Count (TMC)

All microbial colonies were counted to determine TMC—the factor determining mushroom spoilage, deterioration, and non-affordability to human consumption. TMC was assessed on days 0, 1, 2, 3, 4, 5, 6, 7, 8, 9, and 10 at ambient temperature, and days 0, 5, 6, 7, 15, 20, 21, 22, 23, 24, and 27 at 4 °C. The results for microbial loads were reported as colony-forming units per gram (CFU/g) and transformed into log CFU/g values. The categorization of mushroom microbial quality (aerobic microbes) before (day 0) and after storage followed five contamination levels: low: <5.0 log CFU/g, medium: 5.1–6.5 log CFU/g, high: 6.6–8.0 log CFU/g, and very high: >8.1 log CFU/g [38].

### 2.4. Physicochemical Properties and Nutritional Composition Variation in Mushrooms during Storage

The variation in mushroom weight loss, firmness, veil opening, and protein, and carbohydrates contents were evaluated at 0, 1, 2, 3, 4, 5, 6, 7, 8, 9, and 10 days of storage at ambient temperature, and at 0, 5, 6, 7, 15, 20, 21, 22, 23, 24, and 27 days of storage at 4 °C. Weight loss (%) was measured following Equation (2) [39]:

(2) $$Weight\;loss\;\left(\%\right)=\frac{W_{0}-W_{t}}{W_{0}}\times 100$$

where *W*_0_ is the initial mushroom weight and *W_t_* is the mushroom weight at selected times (days) during the storage period.

Mushroom firmness (N) was measured using a TMS-PRO texture analyzer (Food Technology Co., Washington, DC, USA) on a scale of 1–9, where 1 corresponds to “very soft” and 9 to “very firm” [39]. Mushrooms were punched at a 6 mm diameter, 3 mm depth, and with a crosshead speed of 20 mm min^–1^. Veil opening (%) was calculated following Equation (3) [39]:

(3) $$Veil\;opening\;\left(\%\right)=\frac{V_{t}-V_{f}}{V_{t}}\times 100$$

where *V_t_* is the total number of mushroom veils and *V_f_* is the number of open mushroom veils.

Protein and carbohydrate contents are among the most important nutritious contents in mushrooms; thus, their variation during mushroom storage is an essential tool for the assessment of quality deterioration. Protein and carbohydrates contents (% FW) in mushrooms were estimated using the macro-Kjeldahl (N × 4.38), and Anthrone methods, respectively [12].

### 2.5. Statistical Analysis

Statistical modeling, optimization calculations, and graphical representations were performed using the Design Expert (Version 11, StatEase Corp, Minneapolis, MN, USA) software package. Several statistical tests such as analysis of variance (ANOVA), a partial F-test for individual terms, and residuals analysis were performed within the RSM modeling. The determination of coefficients and effects of individual linear, quadratic, and interaction terms were assessed to draw ANOVA results. F-value was calculated at probabilities of 0.001, 0.01, 0.05, or 0.1 to determine the statistical degrees of significance in all terms of the polynomial model. Keeping the variable constant at the center point (0) and varying the other two variables within the experimental range allows for building three-dimensional bi-plots for both atmospheric temperatures of storage (ambient temperature and at 4 °C) (a total of six bi-plots).

## 3. Results and Discussion

### 3.1. Evaluation of RSM Models and Their Performance for Mushroom Shelf-Life Extension during Storage at Ambient Temperature

The optimization of mushroom shelf-life (response variables) was successfully developed using a CCD design. The studied packaging conditions and OLPR/SCG rates affected differently the shelf-life of mushrooms. Measured and predicted values of M_1_, M_2_, and M_3_ shelf-life (days) during storage at ambient temperature are shown in Table 2, based on the designed CCD matrix.

The type of packaging plays a major role in the determination of fresh mushroom shelf-life [40]. Additionally, the type of growing mixture naturally may affect the shelf-life of freshly harvested mushrooms. This has been investigated throughout the current study. Among unpacked mushrooms, those grown on WS substrate had the shortest shelf-life (3.0 days), while those grown on low rates of OLPR (0.3) and SCG (0.3) lasted 4.0–4.3 days more without any sign of microbial spoilage and deterioration (no green mold formation or browning of the mushrooms’ cuticle). A rate of 0.33 OLPR/SCG in *P. ostreatus* substrate showed the longest shelf-life for unpacked–produced mushrooms (4.7 days). All unpacked mushrooms already grown on substrates containing OLPR and/or SCG had an extended shelf-life of 0.3–1.7 days compared to control (WS) mushrooms. Choi and Kim [41] reported a general 1.0–2.0 days-shelf-life for freshly harvested *P. ostreatus* mushrooms stored at ambient temperature. The current findings denoted a 1.5–4.3-fold longer shelf-life for all unpacked oyster mushrooms stored at ambient temperature compared to the aforementioned study. The short shelf-life of fresh oyster mushrooms left at ambient temperature is mainly attributed to their high respiration and transpiration rates, in addition to the lack of protective cuticles on their skin [42]. This normally results in higher water loss and susceptibility to microbial spoilage. Factors such as wilting, browning, and stipe elongation indicated the limit of *P. ostreatus* shelf-life [43], in addition to mold formation, unpleasant odor, and high-water liberation simulating the rapid depletion of nutrients found in the mushrooms’ fruiting body. When packed in polyethylene plastic bag packaging (PBP), control mushrooms had a shorter shelf-life (by 0.3–0.4 days) compared to those grown on substrates containing OLPR and an extended shelf-life (by 0.3–0.8 days) compared to those grown on substrates enclosing SCG rates. On the other hand, a mixture of OLPR and SCG in low and high rates (0.17 and 0.33, respectively) had a longer shelf life of 0.5–1.2 days compared to WS using the same type of packaging. Among all PBP mushrooms, those grown at a high rate of OLPR/SCG (0.33) had the best and most extended shelf-life (9.1 days). Singh et al. [23] reported that storing mushrooms in plastic bags would increase the relative humidity inside the package during the storage period, thus reducing their shelf-life. In the current experiment, the small opening left in the PBP assured an atmosphere closely similar to that in modified atmospheric packaging (MAP), which enables gas exchange inside the package, thus extending the mushrooms’ shelf-life [44].

Similar to PBP, using polypropylene vacuum bag packaging (VBP) showed a longer shelf-life of mushrooms grown on substrates containing OLPR (by 0.4–2.3 days) and a shorter shelf-life of those grown on substrates containing SCG (by 0.4–2.0 days) compared to control mushrooms. An extension of oyster mushroom shelf-life was observed when they were already grown on mixtures of OLPR and SCG (an extension of 0.4–1.0 days) compared to the control. Generally, mushrooms packed in VBP at ambient temperature had a shorter shelf-life of 0.9–2.2 days compared to PBP ones. The interesting and unforeseeable point is that oyster mushrooms grown on substrates with a low rate of OLPR (0.3) had an extended shelf-life of 0.9 days when packed in VBP compared to PBP. Moreover, all mushrooms grown on mixtures of WS and OLPR had an initial moisture content of 87.5–88.0%, which is comparable to the control (87.7%). This suggests that the initial moisture content of these mushrooms did not play a large role in hastening or delaying their deterioration. It also suggests that the low rate of OLPR may have played a role in the reduction in oyster mushroom respiration, delaying the liberation of its water content and thus leading to more microbial spoilage in VBP as compared to PBP. It is a strange and unique observation at ambient temperature that needs further investigation. On the other hand, the shorter shelf-life of mushrooms grown on low and high rates of SCG compared to control ones was mainly related to the higher moisture content of the former in comparison with the latter (90.04%, 91.01, and 87.7%, respectively). It was well reported that increased moisture content in fresh mushrooms is one of the major causes of their fast deterioration [34]. Therefore, the search for cost-effective methodologies should seek the long-term preservation of these mushrooms. *P. ostreatus* grown on substrates containing low and high rates of OLPR/SCG had lower moisture contents (81.46% and 80.5%, respectively) compared to control ones (87.7%). This explains the significant extension of the former’s shelf-life even without any pre-treatment prior to storage. The following model equations along with the coefficients of M_1_ shelf-life, M_2_ shelf-life, and M_3_ shelf-life at ambient temperature can be predicted.

(4) $$Y_{1}=2.995+6.900\;X_{1}+7.370\;X_{2}+0.019\;X_{1}X_{2}-2.413\;X_{1}^{2}-9.889\;X_{2}^{2}\;$$

(5) $$Y_{2}=3.031+7.545\;X_{1}+3.529\;X_{2}-1.996\;X_{1}X_{2}-2.770\;X_{1}^{2}-3.532\;X_{2}^{2}\;$$

(6) $$Y_{3}=3.025+7.569\;X_{1}+5.543\;X_{2}-1.399\;X_{1}X_{2}-2.816\;X_{1}^{2}+1.004X_{2}^{2}$$

Results of the mathematical quadratic models (Table 3) showed that the independent variables, packaging type (*X*_1_) and OLPR, SCG, OLPR/SCG rates (*X*_2_), were significant determinants (*p* < 0.05) of M_1_, M_2_, and M_3_ shelf-life (*Y*_1,_ *Y*_2_, and *Y*_3,_ respectively). All quadratic models (*p* < 0.0001), means of *Y*_1_, *Y*_2,_ and *Y*_3_ (*p* < 0.05), and interactions among independent variables determining *Y*_2_ and *Y*_3_ (*p* < 0.05) were significant, having *p* < F values. The non-significant lack of fit (LoF) for *Y*_1_, *Y*_2_, and *Y*_3_ (*p* > 0.05) and low PRESS values (9.26, 1.04, 0.77, respectively) showed the strength of the quadratic models that were constructed. High *R*^2^ (0.98, 0.99, 0.99), adjusted *R*^2^ (0.97, 0.99, 0.99), predicted *R*^2^ (0.87, 0.97, 0.98), and adequate precision (20.29, 37.13, 53.71) were noted. Based on these findings, the model equations obtained could be successfully used to predict the shelf-life of M_1_, M_2_, and M_3_.

**Figure 2:**
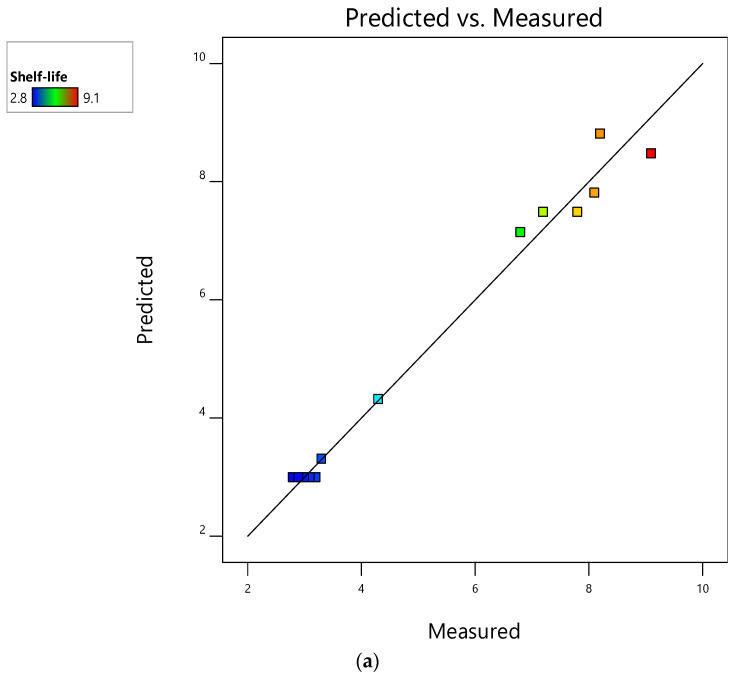

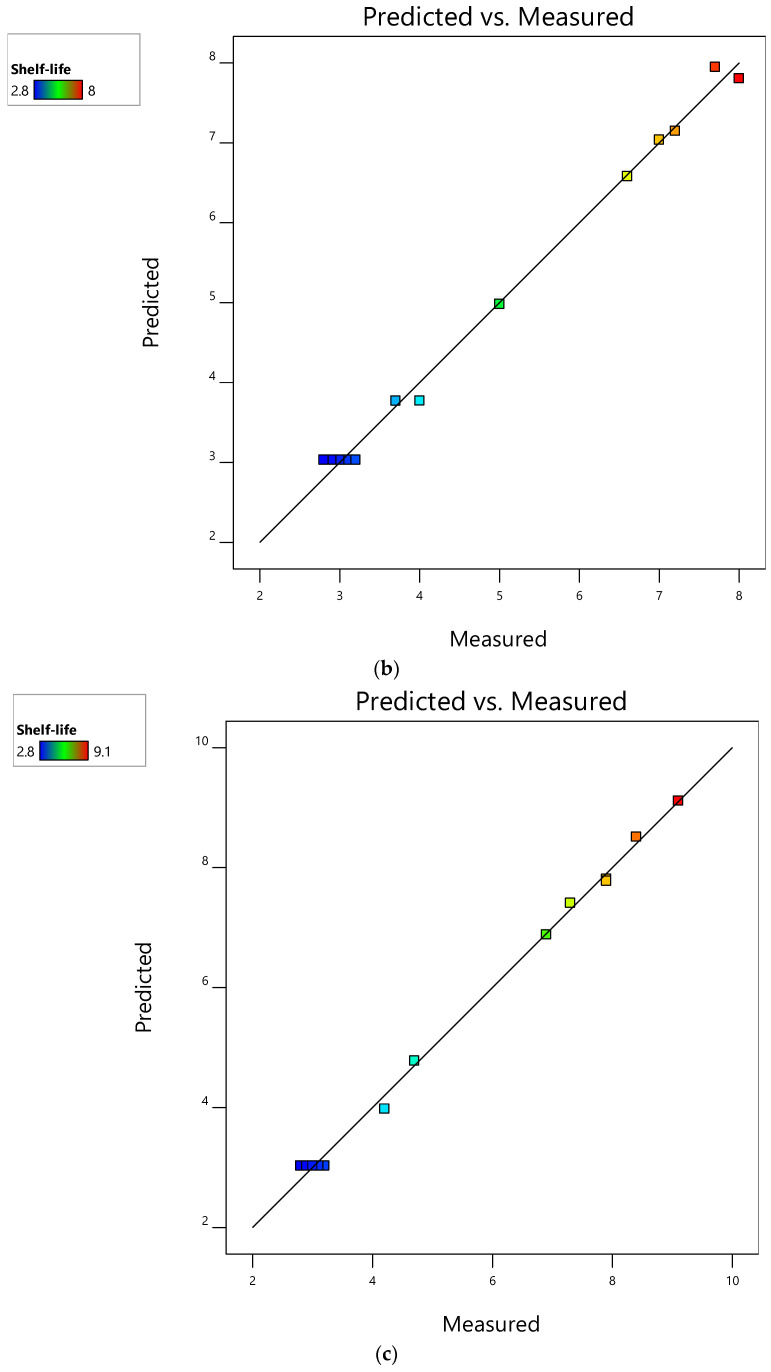
Measured vs. predicted bi-plots for shelf-life extension of mushrooms stored at ambient temperature: (**a**) M_1_ (mushrooms of WS and OLPR mixtures), (**b**) M_2_ (mushrooms of WS and SCG mixtures), and (**c**) M_3_ (mushrooms of WS and OLPR/SCG mixtures).

Mushroom shelf-life is an important factor in determining their quality and marketability for human consumption [45]. Therefore, predicting this response is well demanded to overcome and trigger any mold formation and/or microbial spoilage. Figure 2 shows very few differences among measured and predicted response variable values, adjusted and predicted *R^2^*, and adequate precision.

The current findings outline the usefulness of the constructed models in the prediction and optimization of the shelf-life of M_1_, M_2_, and M_3_ during storage at ambient temperature. This point was already observed in Table 2 where the shortest and longest shelf-life for M_1_, M_2_, and M_3_ were homogenously detected in both measured and predicted values. RSM was successfully used to optimize the packaging material that preserves the most nutritional composition and microbiological quality of *Pleurotus ostreatus* [46]. Additionally, RSM proved to be successful in modeling and optimizing vacuum drying of horticultural crops, including shiitake mushrooms and konjac vegetables [47,48]. These studies encourage further investigation of substrate effect on mushroom shelf-life grown on agro-industrial residues.

### 3.2. Interactive Effect of Factors on Shelf-Life of Mushrooms Stored at Ambient Temperature

The shelf-life of M_1_, M_2_, and M_3_ (responses) during storage at ambient temperature was closely affected by the packaging type adopted and the rates of OLPR, SCG, and OLPR/SCG. Both independent variables (factors) had a *p*-value < 0.05 over the models’ performance. Figure 2 shows the surface plots where the interactive effect of packaging type and OLPR, SCG, and OLPR/SCG rates is detectable.

### 3.3. Optimization of Factors for Extended Shelf-Life of Mushrooms Stored at Ambient Temperature

**Figure 3:**
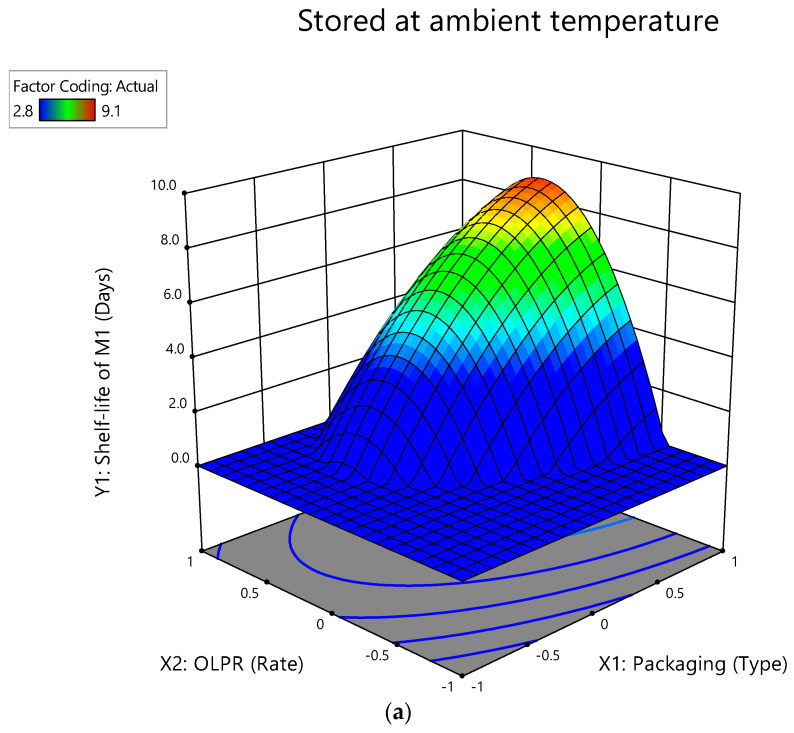

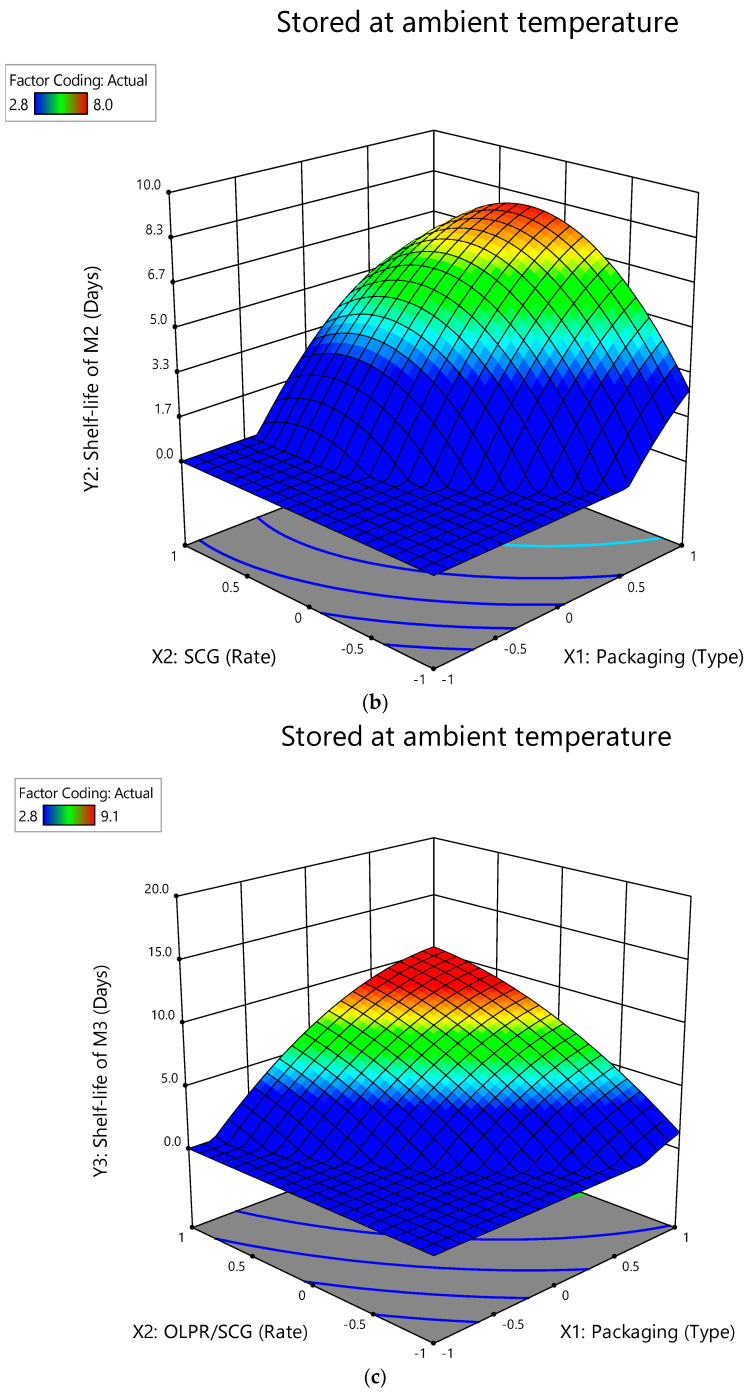
Three-dimensional surface plots for the interactive effect of packaging type and OLPR, SCG, and OLPR/SCG rates on shelf-life during storage at ambient temperature: (**a**) M_1_ (mushrooms of WS and OLPR mixtures); (**b**) M_2_ (mushrooms of WS and SCG mixtures); and (**c**) M_3_ (mushrooms of WS and OLPR/SCG mixtures).

The longest shelf-life of mushrooms is presented as dark red (Figure 3), corresponding to the most suitable packaging type and OLPR, SCG, and OLPR/SCG rates.

In order to maximize the shelf-life of stored mushrooms (responses), adequate factors must be adjusted purposely. The optimization process allows for extending the mushroom’s shelf-life taking into consideration the preservation of the product’s quality in a cost-effective manner. This methodology provides optimization of independent variables (packaging type and OLPR, SCG, and OLPR/SCG rates following a determined range) on the desired value of each response (maximum shelf-life of M_1_, M_2_, and M_3_). The mathematical models predicted the best packaging type and optimum OLPR rate as PBP and 0.289, respectively, for a maximized shelf-life of M_1_ (9.18 days) during storage at ambient temperature (Table 4). M_2_ shelf-life was maximized (8.15 days) using PBP and a rate of SCG equal to 0.23. An OLPR/SCG rate of 0.303 and PBP was enough to maximize M_3_ shelf-life (9.12 days). These findings suggest the efficiency of PBP, low rates of OLPR and SCG, and high OLPR/SCG rate in extending mushroom shelf-life when stored at ambient temperature. The optimum shelf-life is obtained through the addition at a rate of 0.289 of OLPR to the growing substrate, pointing out the positive effect of OLPR on mushroom shelf-life. In addition, the optimum shelf-life extension is achieved when having a combination of OLPR and SCG in the mushroom substrate. This indicates the beneficial incorporation of this combination of agro-industrial residues in *Pleurotus ostreatus* substrate to extend mushroom shelf-life. Additional response values (some being satisfactory and some not satisfactory) were also predicted by the models, but the ones presented herein were considered the most satisfactory according to the same constructed models. The verification of these predictions was assessed by performing separate validation experiments at optimum conditions. Both predicted and experimental values matched, indicating the success of RSM in the optimization of factors (packaging type and OLPR, SCG, and OLPR/SCG rates) for best responses (M_1_, M_2_, and M_3_ shelf-life). Subramaniam et al. [49] depicted a 1.0–3.0 day shelf-life for shiitake mushrooms grown on lignocellulosic materials, when stored at ambient temperature, packed or not. They also outlined that after 3 days of storage, the incidence of microbial colonies increases tremendously. Moreover, Xiao and Zhang [50] grew oyster mushrooms (known as ping gu mushroom in China) on wheat straw substrate. They also reported a 1.0–3.0 day shelf-life with MAP under ambient temperature, which is far below the average shelf-life obtained with PBP in the present study (7.2–9.1 days). After this period, the authors noted that fast microbial spoilage occurs. Sebaaly et al. [51] mentioned 1.0–3.0 day shelf-life for button mushrooms when stored unpacked at ambient temperature, and a 4.0–6.0 day shelf-life when packed in paper bags in the same atmospheric condition. These periods are also shorter than our findings. This outlines the role of agro-industrial residues used (OLPR and SCG separately or combined) in the extension of fresh mushrooms during storage.

### 3.4. Evaluation of RSM Models and Their Performance for Mushroom Shelf-Life Extension during Storage at 4 °C

As performed during ambient temperature storage, the optimization of mushroom shelf-life (response variables) was successfully developed during storage at 4 °C using CCD design. The studied packaging conditions and OLPR/SCG rates affected differently the shelf-life of mushrooms when stored at 4 °C. Measured and predicted values of M1, M2, and M3 shelf-life (days) based on the designed CCD matrix are shown in Table 5.

The adopted agro-industrial residues influenced differently the shelf-life of mushrooms stored at 4 °C. Among unpacked mushrooms and similarly to those stored at ambient temperature, WS mushrooms had the shortest shelf-life (5.0 days). The use of whole mushrooms helped to extend their shelf-life during storage at low temperatures and reduced their respiration and transpiration rates. Mushrooms grown on low rates of OLPR (0.3) and SCG (0.3) lasted longer by 2.1 and 1.8 days, respectively, compared to control mushrooms, and by 0.7 and 0.6 days, respectively, in comparison with those grown at high OLPR (0.7) and SCG (0.7) rates. Mushrooms grown on combined rates of OLPR and SCG had the longest shelf-life period with an increase of 2.2–2.6 days in comparison with control mushrooms. The longest shelf-life among unpacked mushrooms (7.6 days) was observed for those grown on a high OLPR/SCG rate (0.33). This points out the importance of mixing both agro-industrial residues for an extended shelf-life of *P. ostreatus* mushrooms at low-temperature storage, even without any packaging or processing technique. Average shelf-life of 8.0–11.0 and 4.0–6.0 days were earlier reported when fresh oyster mushrooms were stored at freezing and low temperatures (0 and 5 °C), respectively [40,52]. The current study outlined an average shelf-life of 5.0–7.6 days when mushrooms were stored unpacked at 4 °C, being in line with the aforementioned findings of Villaescusa and Gil [52] at low temperatures. The extension of mushroom shelf-life at a low temperature is promoted by the slowed metabolic and heat rates, in addition to retardation of the deterioration process [53].

Generally, PBP and VBP are widely used as mushroom packaging materials for storage at low temperatures, including refrigeration and freezing [46]. When packed in PBP, control mushrooms had a shorter shelf-life of 1.6–2.7, 1.7–1.9, and 3.0–4.6 days compared to mushrooms grown at OLPR, SCG, and OLPR/SCG rates, respectively. Among all PBP mushrooms, the longest shelf-life was observed for those grown on a combination of OLPR and SCG (24.8 days at a high rate of 0.33). These findings are in line with the previous reports of Ajayi et al. [46], who acknowledged the role of high-density polyethylene bags in the quality preservation of *P. ostreatus* mushrooms. The small opening left in the PBP constituted a modified atmospheric packaging (MAP), which helped in the control of humidity inside the selected bags [54].

Several studies outlined the role of packaging and agro-industrial residues in the extension of fresh mushrooms shelf-life at low temperature. For instance, different MAP levels were tested by Fu et al. [55] on *Trichloma matsutake* mushrooms stored at 4 °C. They outlined the mushroom’s shelf-life in the range of 10–18 days, which is shorter than observed in this study and that above this range, the incidence of microbial colonies increased tremendously by several fold. Furthermore, Sebaaly et al. [51] investigated the role of banana residues in the extension of fresh button mushrooms shelf-life at ambient temperature and at 4 °C. They depicted a shelf-life of 9–10 days when mushrooms grown on banana residues were stored in paper bags in the refrigerator. After this period, high water loss and microbial spoilage occurred, making stored mushrooms inconsumable. Although this period was much less than reported in the current study, combined factors, i.e., packaging type, type of agricultural residues, and mushroom species, might be behind such result. Moreover, Xiao et al. [56] stored fresh *P. ostreatus* mushrooms in MAP at 6 °C after dipping in different chemical treatments. They outlined a shelf-life period of 13 days, being therefore in the range stated by Fu et al. [55] but less than found in the present study. This confirms that the agro-industrial residues are the real factor behind the increased shelf-life period. Mushrooms farmers are therefore invited to use such agro-industrial residues for mushroom growing instead of the use of chemical treatments to extend their product’s shelf-life (being actually not very efficient) that might harm the consumer.

Similar to PBP, the use of VBP resulted in an extended shelf-life of mushrooms grown on substrates containing OLPR, SCG, or combined agro-industrial residues. Lower OLPR (0.3) and SCG (0.3) rates induced a longer shelf-life of mushrooms rather than higher rates (0.7 OLPR and SCG) (extension by 0.9 and 0.4 days, respectively). Generally, mushrooms grown at both low and high rates of OLPR and SCG lasted longer by 1.1–2.2 and 1.1–1.5 days, respectively, compared to WS mushrooms. It is noteworthy that mushrooms grown at high rates of OLPR and SCG had a similar shelf-life (22.8 days). Furthermore, a combination of OLPR and SCG in the initial substrate resulted in mushrooms with a range of 2.6–4.4 days longer shelf-life, being the most appropriate for the extension of the latter and therefore delayed consumption’s expiry date. From the marketability point, VBP did not seem to extend much the shelf-life of mushrooms when stored at 4 °C in comparison with PBP. However, this short extension could be beneficial in rural areas with low income and thus low purchasing power of consumers. Additionally, current findings contradicted the public knowledge on the effect of VBP on hastening the deterioration of fresh mushrooms at low-temperature storage. Kang et al. [57] stored mushrooms at low temperatures in both full-vacuumed bags and semi-vacuumed ones; they reported that the former led to color deterioration while the latter was a suitable type of packaging. The current findings may directly lead to the simulation of a positive effect of OLPR and SCG on the calibration of mushroom respiration and transpiration rates and thus the extension of *P. ostreatus* shelf-life.

The following provides the model equations along with the coefficients by which M_1_ shelf-life, M_2_ shelf-life, and M_3_ shelf-life during storage at 4 °C can be predicted:

(7) $$Y_{1}=5.495+22.100\;X_{1}+10.525\;X_{2}+0.323\;X_{1}X_{2}-7.014\;X_{1}^{2}-13.265\;X_{2}^{2}$$

(8) $$Y_{2}=5.520+22.014\;X_{1}+7.111\;X_{2}+0.431\;X_{1}X_{2}-6.997\;X_{1}^{2}-8.543\;X_{2}^{2}$$

(9) $$Y_{3}=5.510+22.526\;X_{1}+12.973\;X_{2}+3.871\;X_{1}X_{2}-7.290\;X_{1}^{2}-17.853X_{2}^{2}$$

Results of the mathematical quadratic model (Table 6) showed that the independent variables, packaging type (*X*_1_) and OLPR, SCG, OLPR/SCG rates (*X*_2_), were significant determinants (*p* < 0.05) of M_1_, M_2_, and M_3_ shelf-life (*Y*_1_, *Y*_2_, and *Y*_3_, respectively) during storage at 4 °C. All quadratic models (*p* < 0.0001), means of *Y*_1_, *Y*_2_, and *Y*_3_ (*p* < 0.05), and interactions between independent variables determining *Y*_3_ (*p* < 0.05) were significant, having *p* < F values. The non-significant lack of fit (LoF) for *Y*_1_, *Y*_2_, and *Y*_3_ (*p* > 0.05) and low PRESS values (3.33, 3.29, 7.97) showed the strength of the quadratic models that were constructed. High *R^2^* (0.99, 0.99, 0.99), adjusted *R*^2^ (0.99, 0.99, 0.99), predicted *R*^2^ (0.99, 0.99, 0.99), and adequate precision (70.90, 74.41, 70.81) were noted. Based on these findings, the model equations can be successfully used to predict the shelf-life of M_1_, M_2_, and M_3_.

**Figure 4:**
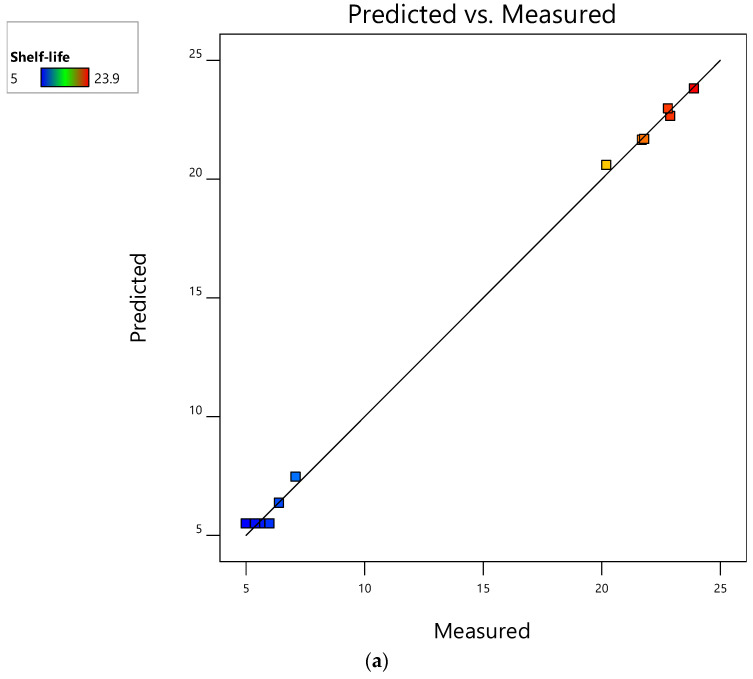

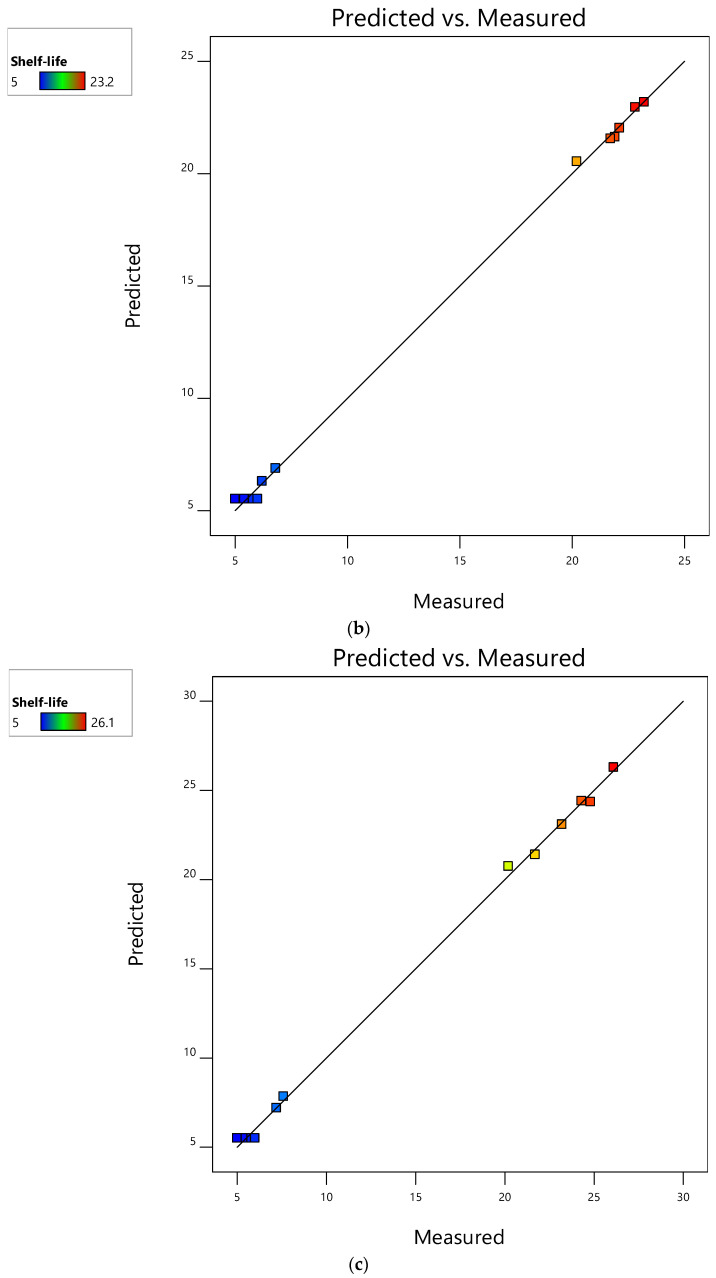
Measured vs. predicted bi-plots for shelf-life extension of mushrooms stored at 4 °C: (**a**) M_1_ (mushrooms of WS and OLPR mixtures); (**b**) M_2_ (mushrooms of WS and SCG mixtures); and (**c**) M_3_ (mushrooms of WS and OLPR/SCG mixtures).

Figure 4 shows minimal differences among the values of measured and predicted response variables, adjusted and predicted *R*^2^, and adequate precision. These findings outline the usefulness of the constructed models in the prediction and optimization of the shelf-life of M_1_, M_2_, and M_3_ during their storage at 4 °C. This point was already observed in Table 5, where the shortest and longest shelf-life of M_1_, M_2_, and M_3_ were homogenously detected in both measured and predicted values. The prediction of fresh mushroom shelf-life during cold storage at 4 °C via RSM seems unique. The main concern of studies is mainly concentrated on freeze-drying, hot-drying, or high-pressure steamed mushrooms, and predicting the most suitable methodology between the aforementioned for extending mushroom shelf-life and improving their nutritional composition [37,58].

### 3.5. Interactive Effects of Factors on Shelf-Life of Mushrooms Stored at 4 °C

The shelf-life of M_1_, M_2_, and M_3_ (responses) during storage at 4 °C was closely affected by the packaging type adopted and the rates of OLPR, SCG, and OLPR/SCG. Both independent variables (factors) had a *p*-value < 0.05 over the models’ performance. Figure 4 shows the surface plots where the interactive effect of packaging type and OLPR, SCG, and OLPR/SCG rates is detectable.

### 3.6. Optimization of Factors for Extended Shelf-Life of Mushrooms Stored at 4 °C

**Figure 5:**
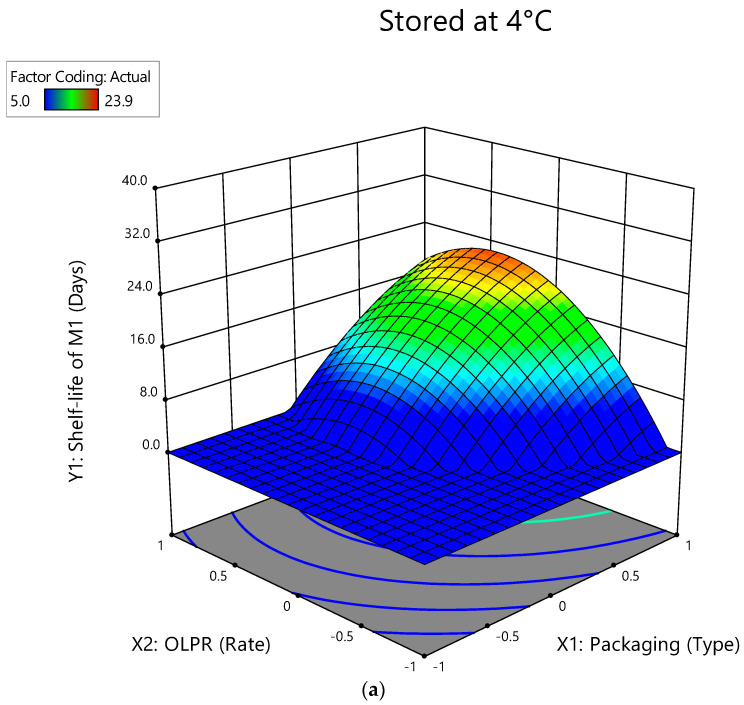

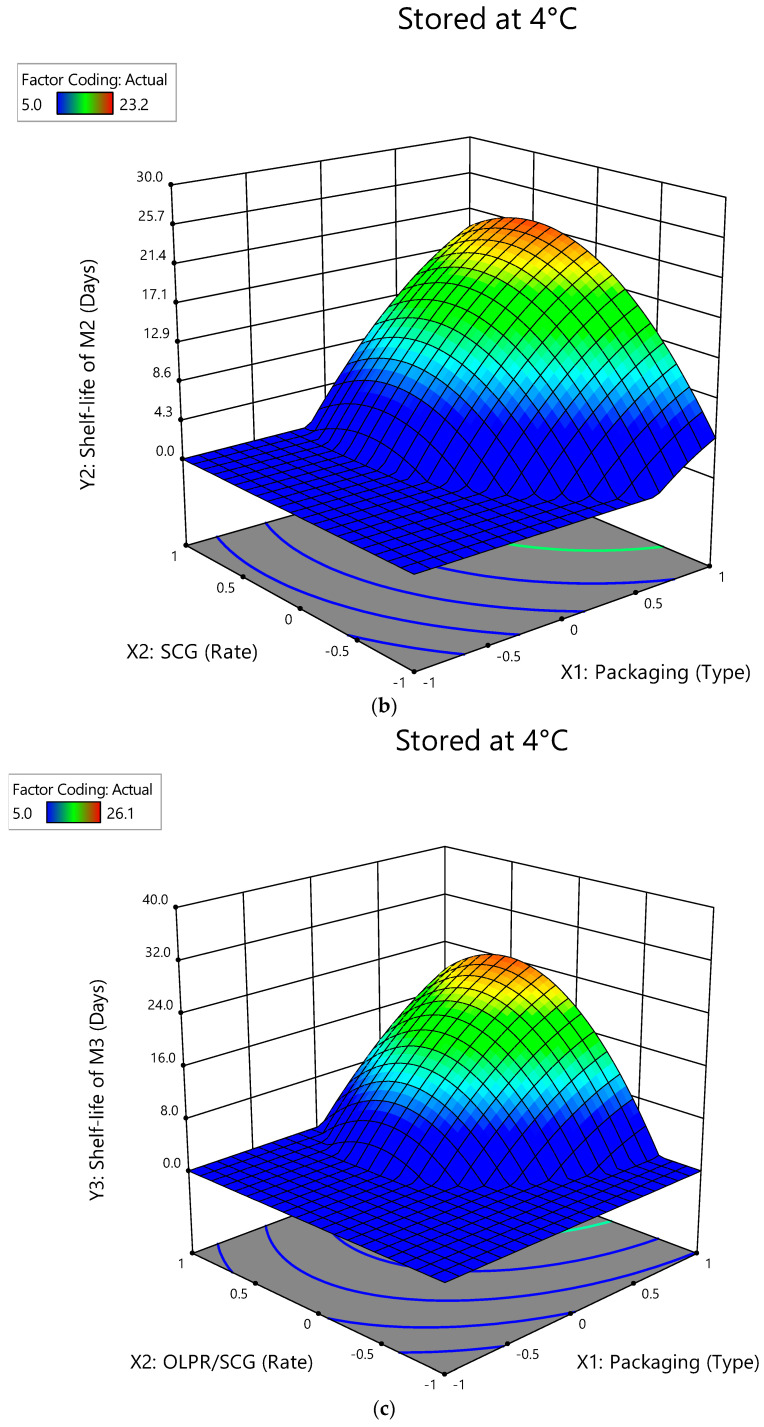
Three-dimensional surface plots for the interactive effect of packaging type and OLPR, SCG, and OLPR/SCG rates on shelf-life (days) during storage at 4 °C: (**a**) M_1_ (mushrooms of WS and OLPR mixtures); (**b**) M_2_ (mushrooms of WS and SCG mixtures); and (**c**) M_3_ (mushrooms of WS and OLPR/SCG mixtures).

The longest shelf-life of mushrooms is presented in red (Figure 5), corresponding to the most suitable packaging type and OLPR, SCG, and OLPR/SCG rates.

The mathematical models predicted the best packaging type and optimum OLPR rate as VBP and 0.27, respectively, for a maximized shelf-life of M_1_ (24.90 days) during storage at 4 °C (Table 7). M_2_ shelf-life was maximized (23.67 days) using the same type of packaging and a rate of SCG equal to 0.13. An OLPR/SCG rate equal to 0.22 and VBP were enough to maximize M_3_ shelf-life to 26.26 days. These findings suggest the efficiency of VBP, and low rates of OLPR, SCG, and OLPR/SCG in the substrate in the extension of mushroom shelf-life when stored at 4 °C. The optimum shelf-life is obtained through the addition of a rate equal to 0.22 of OLPR/SCG to the growing substrate, pointing out the positive effect of combined OLPR and SCG on mushroom shelf-life when stored at 4 °C. In addition, a notable shelf-life extension is achieved when having independent rates of OLPR and SCG in the mushroom substrate. This again points out the importance of incorporating these agro-industrial residues in *P. ostreatus* substrate and thus the extension of produced mushroom shelf-life. Additionally, response values (some being satisfactory and some not satisfactory) were predicted by the models, but the ones presented herein were considered the most satisfactory according to the same constructed models. The verification of these predictions was assessed by performing separate validation experiments at optimum conditions. Both predicted and experimental values matched, indicating the success of RSM in the optimization of factors (packaging type and OLPR, SCG, and OLPR/SCG rates) for best responses (M_1_, M_2_, and M_3_ shelf-life). Sebaaly et al. [51] found that *Agaricus bisporus* mushrooms bagged in paper bags and stored at low temperatures had a minimal shelf-life of 7 and 10 days when grown on horse manure substrate and banana wastes, respectively. This shelf-life period is much less than observed in the current study, probably due to a difference in mushroom species and initial substrate. It should be pointed out that a close interrelationship exists among the optimization of substrate and packaging types, mushroom species, and mushroom shelf-life, mostly corroborated by their decreased moisture content and the capacity of the package to absorb water liberated via respiration and transpiration during storage.

### 3.7. Total Microbial Count (TMC)

All mushrooms stored at ambient temperature showed a low contamination level (TMC < 5.0 log CFU/g) on days 0 and 1 (Table 8). On day 2, all unpacked mushrooms showed a medium level of contamination ranging between 5.4 and 6.5 log CFY/g, while packed ones were associated with a low contamination level (3.1–4.9 log CFU/g). On day 3, unpacked mushrooms showed a high level of contamination (7.1–7.8 log CFU/g) while the majority of packed mushrooms (83.3%) still showed a low contamination level (except mushrooms grown on mixtures of WS, OLPR, and SCG with VBP). On day 4, half of the unpacked mushrooms showed a high level of contamination (7.6–8.0 log CFU/g) while the other half depicted a very high level of contamination (8.2–8.6 log CFU/g). Whereas, 77.8% and 88.9% of mushrooms in PBP and VBP still outlined low and medium contamination levels, respectively. By day 5, all unpacked mushrooms were highly contaminated (8.2–8.6 log CFU/g), whereas the majority (8.3.3%) of packed mushrooms (except those in VBP grown on mixtures of WS, OLPR, and SCG) showed a medium contamination level. On day 6, 83.3% of packed mushrooms were highly (77.7%) or very highly (5.6%) contaminated, whereas mushrooms grown on mixtures of WS, OLPR, and SCG in PBP and others grown on a mixture of WS and low OLPR rate still showed a medium contamination level. By day 7 and beyond, all packed mushrooms were either highly or very highly contaminated. Only mushrooms grown on mixtures of WS and high rates of OLPR/SCG and those grown on mixtures of WS and a low OLPR rate did not show a very high contamination level until day 10 (8.3–8.4 log CFU/g).

Schill et al. [38] reported a low level of contamination (average 4.2 log CFU/g) at day 0 storage of *P. ostreatus*, which is even higher than the current findings. Additionally, Wang et al. [59] noted that TMC at the harvest level of *P. ostreatus* was around 3.5 log CFU/g, which is comparable with findings obtained herein or even slightly higher. In the same context, Siyoum et al. [60] reported that mushrooms with microbial counts ranging between 4.0 and 5.0 log CFU/g at harvest time are very safe. It was depicted that a combination of factors such as water content and microbial load affects directly the stability of mushrooms during storage [61]. This is well the case observed in the current study, where mushrooms with lower moisture content (mixtures containing OLPR and/or OLPR/SCG) delayed the multiplication of microbial populations and thereby deterioration compared to control. Further, the raw material quality (i.e., substrate type), type of processing (i.e., packaging types), and storage conditions (i.e., refrigerated or not) are determining factors of mushroom quality (low–medium TMC) [42]. This hypothesis is well confirmed by the current findings, where the presence of OLPR combined or not with SCG in initial growing substrates resulted in mushrooms with low–medium TMCs for a relatively long time (3.0–4.0 days) compared to WS mushrooms in PBP and VBP.

Table 9 shows TMCs on mushrooms stored at 4 °C. Results revealed that all unpacked mushrooms had low (3.4–4.1 log CFU/g) and medium (5.3–6.5 log CFU/g) levels of contamination at days 0 and 5, respectively. Two-thirds of these mushrooms were highly contaminated (6.9–7.9 log CFU/g) and the remaining third were very highly contaminated (8.4 log CFU/g) by day 6. By day 8, all unpacked mushrooms stored at 4 °C were very highly contaminated (>8.1 log CFU/g). On the other hand, all packed mushrooms showed a low contamination level (1.0–4.3 log CFU/g) during the first seven days of storage being even lower than the average TMC reported by Schill et al. [38] on *P. ostreatus* after seven days of storage (5.2 log CFU/g). By day 15, two-thirds of packed mushrooms outlined a medium level of contamination (5.8–6.5 log CFU/g) while the remaining third (main mushrooms grown on mixtures of WS, OLPR, and SCG) was still associated with a low level of contamination (4.2–4.9 log CFU/g). Mushrooms of the latter denoted a medium level of contamination (5.1–6.2 log CFU/g) by day 20, whereas the majority of the remaining mixtures presented a high contamination level (7.4–7.9 log CFU/g). In further days, the majority of PBP and VBP mushrooms were high to very highly contaminated (6.6–8.8 log CFU/g).

Generally, high TMCs were reported to be associated with the presence of *Pseudomonas* spp. and *E. americana* populations [62,63]. Several studies outlined the safety of mushrooms with bacterial loads ranging between 5.2 and 12.4 log CFU/g [60] or between 7 and 9 log CFU/g [63]. Based on that, at ambient temperature all unpacked mushrooms could be consumable until day 4; whereas those incorporated in PBP and VBP can withstand until days 9 and 6, respectively. Mushrooms stored at 4 °C could be consumable until day 7 when unpacked and day 22 when packed. However, for greater safety, the classification of Schill et al. [38] would be more suitable and convincing as mushrooms with a contamination level > 8.1 log CFU/g were ranked unfavorable by these authors.

### 3.8. Physicochemical Properties and Nutritional Composition Variation in Mushrooms during Storage

M_3_B and M_3_C showed the lowest weight loss when stored unpacked at ambient temperature (with a range of 26.1–26.5%) (Table S1; Supplementary Materials). However, mushrooms are still considered consumable until they lose around 5% of their weight [64]. Therefore, M_3_B and M_3_C can still be consumable until day 4, which confirms the aforementioned shelf-life assessment and TMC analysis. Similarly, M_3_C and M_1_B showed the lowest WL (4.9% at day 9) when stored in PBP and VBP at ambient temperature, respectively. Unpacked M_1_B, M_3_B, and M_3_C had the highest firmness at the day of harvest (9.0) and maintained acceptable values (3.5–3.7) until day 4 at ambient temperature. According to López-Gómez et al. [65], mushrooms exhibiting a firmness value greater than 3.0 are considered acceptable for consumption. M_3_B and M_3_C, and M_1_B stored in PBP and VBP, respectively, retained consumable firmness values (3.3.–3.6) until day 8. Below a 3.0 firmness value, mushrooms are considered soft, non-marketable, and undesired by consumers. Sami et al. [39] considered a 70% veil opening as high and a sign of deterioration. Accordingly, unpacked M_3_C or stored in PBP at ambient temperature did not reach this percentage until days 5 and 10, respectively, whereas M_1_B, M_3_B, and M_3_C reached this percentage at day 8 when stored in VBP at ambient temperature. Protein and carbohydrates contents in all mushrooms decreased by 54.8–62.1%, and 28.6–56.45%, respectively, after 10 days of storage at ambient temperature, regardless the type of packaging and used substrates. M_1_B maintained the highest protein content during storage (2.0–2.1%) whereas M_2_C recorded the highest carbohydrates content (5.1–5.5%), both regardless the type of packaging used. Although such results reveal the reduction in mushroom nutritional value during storage, these values are still very promising and reflect the important role of agro-industrial residues in the maintenance of good quality mushrooms [12].

M_3_C showed the lowest weight loss when stored at 4 °C, regardless the type of packaging (with a range of 5.8–48.1%) (Table S2; Supplementary Materials). Based on the recommendations of Mahajan et al. [64], unpacked M_3_C stored at 4 °C can still be consumable until day 7, which confirms the aforementioned shelf-life assessment and TMC analysis. M_3_C withstood until day 24 (with a WL range of 4.6–4.9%) when stored in PBP and VBP at 4 °C. Sami et al. [39] reported that the weight loss of fresh button mushrooms stored in PBP at 4 °C was around 14.0%, which makes them inconsumable; whereas, the current findings outlined that oyster mushrooms stored under the same conditions can withstand 20 days. This is probably a species-related issue, which controls the respiration rate and enzyme function regulation [66]. At 4 °C, M_3_C had the highest firmness at harvest time (9.0) and during the storage period. This value decreased in unpacked, PBP, and VBP states until reaching the lowest acceptable firmness limit at days 7, 24, and 24, respectively. The firmness of button mushrooms stored in PBP at 4 °C scored a value of 6.5 on day 6 of the storage period [39], which corroborates with the current findings. During storage at 4 °C, M_3_C scored the lowest veil opening with an acceptable range of 60.0–65.0% at day 7 (unpacked) and day 24 (stored in PBP and VBP). Sami et al. [39] reported a 20.0% veil opening when button mushrooms were stored in PBP at 4 °C; whereas, our findings on oyster mushrooms outlined a 2.5-fold-higher percentage in the same storage conditions. Protein and carbohydrates contents in all mushrooms decreased by 56.8–89.7% and 22.7–41.9%, respectively, after 27 days of storage at 4 °C, regardless the type of packaging and substrates used. M_1_B maintained the highest protein content during storage (1.2–2.0%); whereas, M_2_C recorded the highest carbohydrates content (5.2–5.8%), both regardless the type of packaging used. Although such results reveal the reduction in mushroom nutritional value during storage, these values are still very promising and reflect the important role of agro-industrial residues in the maintenance of good quality mushrooms [12] under low-temperature conditions.

## 4. Conclusions

The current findings showed that substrates based on mixtures of WS and OLPR and a combination of WS, OLPR, and SCG increased the shelf-life period of mushrooms at ambient temperature compared to WS. The storage period of mushrooms at 4 °C increased by the incorporation of agro-industrial residues each apart with WS or combined in the growing substrate compared to WS mushrooms. PBP with a small opening ensuring a type of modified atmosphere and VBP, both associated with low rates of OLPR (0.3 OLPR) and SCG (0.3 SCG) and a high rate of OLPR/SCG (0.33 OLPR/SCG), were considered the best conditions for an optimized storage period at ambient temperature and 4 °C, respectively (9.10, 8.00, and 9.10 days; 23.90, 23.20, and 26.10 days, respectively). Until 7 and 15 days, packed (PBP and VBP) mushrooms could withstand (low–medium TMC) when stored at ambient temperature and 4 °C, respectively, with a preference for those grown on OLPR- and OLPR/SCG-based mixtures. The current study is the first to show the close interrelationship between agro-industrial residues in growing substrate and the extension of fresh *P. ostreatus* mushroom shelf-life via an observed and predicted approach using RSM. The physicochemical and nutritional value analysis confirmed the results of the TMC analysis; 0.33 OLPR/SCG PBP-bagged and VBP-bagged mushrooms were the most consumable after 7 and 24 days of storage at ambient temperature and 4 °C, respectively, with acceptable weight loss, firmness, and veil opening. Control mushrooms and 0.7 SCG ones had the highest protein and carbohydrates contents after 10 and 27 days of storage at ambient temperature and 4 °C (2.0–2.1% and 1.2–2.0%; 5.1–5.4% and 5.2–5.8%, respectively). However, all mushrooms grown on agro-industrial residues maintained promising protein and carbohydrates contents, thus outlining the role of these residues in the quality maintenance of stored mushrooms.

Further research is currently underway to investigate the detailed sensorial attributes before and after storage of *Pleurotus ostreatus* mushrooms grown on OLPR and SCG. Further microbial analysis should be performed to classify the detailed mold, yeast, aerobic microbial counts (AMCs), and other microbial communities that primarily limit the shelf-life of *Pleurotus ostreatus* mushrooms. Moreover, a wider range of temperatures and packaging types should be the core of future studies on mushroom shelf-life grown on similar types of agro-industrial residues.

## Figures and Tables

**Table 1:** Actual and coded levels of the experimental variables (factors) for mushroom shelf-life (responses).

Coded Symbol	Variable	Levels		
		−1 (Low)	0 (Medium)	+1 (High)
(*X*_1_)	Packaging type	Vacuum bag packaging (VBP)	No packaging (-)	Plastic bag packaging (PBP)
(*X*_2_)	OLPR rate	0	0.3	0.7
	SCG rate	0	0.3	0.7
	OLPR/SCG rate	0	0.17	0.33

**Table 2:** Measured and predicted response matrix for the shelf life of mushrooms (days) stored at ambient temperature.

Run	Factor Variables		Response Variables					
	Packaging Type (*X*_1_)	OLPR, SCG, OLPR/SCG Rates (*X*_2_)	^d^ M_1_ Shelf-Life (*Y*_1_: Days)		^e^ M_2_ Shelf-Life (*Y*_2_: Days)		^f^ M_3_ Shelf-Life (*Y*_3_: Days)	
			Measured	Predicted	Measured	Predicted	Measured	Predicted
1	0 (^a^-)	−1 (0)	3.00	3.00	3.00	3.03	3.00	3.03
2	0 (-)	0 (0.3 OLPR, 0.3 SCG, 0.17 OLPR/SCG)	4.30	4.32	4.00	3.77	4.20	4.00
3	0 (-)	1 (0.7 OLPR, 0.7 SCG, 0.33 OLPR/SCG)	3.30	3.31	3.70	3.77	4.70	4.78
4	1 (^b^ PBP)	−1 (0)	7.80	7.48	8.00	7.81	7.90	7.78
5	1 (PBP)	0 (0.3 OLPR, 0.3 SCG, 0.17 OLPR/SCG)	8.20	8.81	7.70	7.95	8.40	8.51
6	1 (PBP)	1 (0.7 OLPR, 0.7 SCG, 0.33 OLPR/SCG)	8.10	7.81	7.20	7.15	9.10	9.11
7	−1 (^c^ VBP)	−1 (0)	6.80	7.14	7.00	7.04	6.90	6.90
8	−1 (VBP)	0 (0.3 OLPR, 0.3 SCG, 0.17 OLPR/SCG)	9.10	8.47	6.60	6.58	7.30	7.39
9	−1 (VBP)	1 (0.7 OLPR, 0.7 SCG, 0.33 OLPR/SCG)	7.20	7.48	5.00	4.98	7.90	7.81
10	0 (-)	−1 (0)	3.10	3.00	2.90	3.03	3.10	3.03
11	0 (-)	−1 (0)	2.90	3.00	3.10	3.03	2.90	3.03
12	0 (-)	−1 (0)	3.20	3.00	2.80	3.03	3.20	3.03
13	0 (-)	−1 (0)	2.80	3.00	3.20	3.03	2.80	3.03

^a^- no packaging; ^b^ PBP: plastic bag packaging; ^c^ VBP: vacuum bag packaging; ^d^ M_1_: mushrooms of WS and OLPR mixtures; ^e^ M_2_: mushrooms of WS and SCG mixtures; ^f^ M_3_: mushrooms of WS, OLPR, and SCG mixtures.

**Table 3:** Model variables and ANOVA results for shelf-life of mushrooms (days) stored at ambient temperature.

Parameter	Variable	Sum of Sq.	Mean Sq.	F-Value	*p*-Value	*R* ^2^	Adjusted *R*^2^	Predicted *R*^2^	Adeq. Precision	PRESS
Self-life of ^c^ M_1_ (*Y*_1_)	Model	73.04	14.61	82.15	<0.0001	0.9832	0.9713	0.8753	20.2934	9.26
	^a^ *X*_1_	25.30	25.30	142.30	<0.0001					
	^b^ *X*_2_	3.32	3.32	18.69	0.0035					
	*X*_1_ *X*_2_	0.0002	0.0002	0.0014	0.9712					
	*X*_1_^2^	12.31	12.31	69.24	<0.0001					
	*X*_2_^2^	3.01	3.01	16.92	0.0045					
	Residual	1.24	0.1778							
	Lack of Fit	1.14	0.3816	15.26	0.1118					
Shelf-life of ^d^ M_2_ (*Y*_2_)	Model	50.17	10.03	264.28	<0.0001	0.9947	0.9910	0.9794	37.1381	1.04
	*X*_1_	30.25	30.25	796.93	<0.0001					
	*X*_2_	0.7622	0.7622	20.08	0.0029					
	*X*_1_ *X*_2_	2.52	2.52	66.31	<0.0001					
	*X*_1_^2^	16.23	16.23	427.54	<0.0001					
	*X*_2_^2^	0.3838	0.3838	10.11	0.0155					
	Residual	0.2657	0.0380							
	Lack of Fit	0.1657	0.0552	2.21	0.2294					
Shelf-life of ^e^ M_3_ (*Y*_3_)	Model	71.57	14.31	514.60	<0.0001	0.9973	0.9953	0.9892	53.7123	0.7771
	*X*_1_	30.39	30.39	1092.38	<0.0001					
	*X*_2_	0.3586	0.3586	12.89	0.0089					
	*X*_1_ *X*_2_	0.2370	0.2370	8.52	0.0224					
	*X*_1_^2^	16.73	16.73	601.45	<0.0001					
	*X*_2_^2^	0.0010	0.0010	0.0366	0.0037					
	Residual	0.1974	0.0278							
	Lack of Fit	0.0947	0.0316	1.26	0.3994					

^a^*X*_1_: packaging type; ^b^*X*_2_: OLPR, SCG, OLPR/SCG rates; ^c^ M_1_: mushrooms of WS and OLPR mixtures; ^d^ M_2_: mushrooms of WS and SCG mixtures; ^e^ M_3_: mushrooms of WS, OLPR, and SCG mixtures. Significance is considered when *p*-value < 0.05.

**Table 4:** Optimization results for extended shelf-life (days) of M_1_, M_2_, and M_3_ stored at room temperature using the developed models.

Variable	Target	Optimum Parameter Range
*X*_1_: Packaging (type)	In-range	^a^ PBP (*Y*_1_, *Y*_2_, *Y*_3_)
*X*_2_: OLPR, SCG, OLPR/SCG (rates)	In-range	0.289 (*Y*_1_), 0.23 (*Y*_2_), 0.303 (*Y*_3_)
*Y*_1_: Shelf-life of ^b^ M_1_ (days)	Maximized	9.18
*Y*_2_: Shelf-life of ^c^ M_2_ (days)	Maximized	8.15
*Y*_3_: Shelf-life of ^d^ M_3_ (days)	Maximized	9.14

^a^ PBP: plastic bag packaging; ^b^ M_1_: mushrooms of WS and OLPR mixtures; ^c^ M_2_: mushrooms of WS and SCG mixtures; ^d^ M_3_: mushrooms of WS, OLPR, and SCG mixtures.

**Table 5:** Measured and predicted response matrix for the shelf life of mushrooms (days) stored at 4 °C.

Run	Factor Variables		Response Variables					
	Packaging Type (*X*_1_)	OLPR, SCG, OLPR/SCG Rates (*X*_2_)	^d^ M_1_ Shelf-Life (*Y*_1_: Days)		^e^ M_2_ Shelf-Life (*Y*_2_: Days)		^f^ M_3_ Shelf-Life (*Y*_3_: Days)	
			Measured	Predicted	Measured	Predicted	Measured	Predicted
1	0 (^a^-)	−1 (0)	5.00	5.50	5.00	5.52	5.00	5.51
2	0 (-)	0 (0.3 OLPR, 0.3 SCG, 0.17 OLPR/SCG)	7.10	7.46	6.80	6.89	7.20	7.20
3	0 (-)	+1 (0.7 OLPR, 0.7 SCG, 0.33 OLPR/SCG)	6.40	6.36	6.20	6.31	7.60	7.85
4	+1 (^b^ PBP)	−1 (0)	20.20	20.58	20.20	20.58	20.20	20.75
5	+1 (PBP)	0 (0.3 OLPR, 0.3 OLPR, 0.17 OLPR/SCG)	22.90	22.64	22.10	22.03	23.20	23.09
6	+1 (PBP)	+1 (0.7 OLPR, 0.7 SCG, 0.33 OLPR/SCG)	21.80	21.68	21.90	21.63	24.80	24.36
7	−1 (^c^ VBP)	−1 (0)	21.70	21.64	21.70	21.56	21.70	21.40
8	−1 (VBP)	0 (0.3 OLPR, 0.3 OLPR, 0.17 OLPR/SCG)	23.90	23.80	23.20	23.18	24.30	24.41
9	−1 (VBP)	+1 (0.7 OLPR, 0.7 SCG, 0.33 OLPR/SCG)	22.80	22.96	22.80	22.96	26.10	26.29
10	0 (-)	−1 (0)	5.40	5.50	5.40	5.52	5.40	5.51
11	0 (-)	−1 (0)	5.60	5.50	5.60	5.52	5.60	5.51
12	0 (-)	−1 (0)	5.80	5.50	5.80	5.52	5.80	5.51
13	0 (-)	−1 (0)	6.00	5.50	6.00	5.52	6.00	5.51

^a^-: no packaging; ^b^ PBP: plastic bag packaging; ^c^ VBP: vacuum bag packaging; ^d^ M_1_: mushrooms of WS and OLPR mixtures; ^e^ M_2_: mushrooms of WS and SCG mixtures; ^f^ M_3_: mushrooms of WS, OLPR, and SCG mixtures.

**Table 6:** Model variables and ANOVA results for shelf-life of mushrooms (days) stored at 4 °C.

Parameter	Variable	Sum of Sq.	Mean Sq.	F-Value	*p*-Value	*R* ^2^	Adjusted *R*^2^	Predicted *R*^2^	Adeq. Precision	PRESS
Self-life of ^c^ M_1_ (*Y*_1_)	Model	870.14	174.03	1205.59	<0.0001	0.9988	0.9980	0.9962	70.9096	3.33
	^a^ *X*_1_	259.56	259.56	1798.15	<0.0001					
	^b^ *X*_2_	6.78	6.78	46.96	0.0002					
	*X*_1_ *X*_2_	0.659	0.659	0.5563	0.5210					
	*X*_1_^2^	103.99	103.99	720.38	<0.0001					
	*X*_2_^2^	5.41	5.41	37.49	0.0005					
	Residual	1.01	0.1444							
	Lack of Fit	0.4185	0.1395	0.9425	0.4993					
Shelf-life of ^d^ M_2_ (*Y*_2_)	Model	849.76	169.95	1392.08	<0.0001	0.9999	0.9983	0.9961	74.4103	3.29
	*X*_1_	257.55	257.55	2109.56	<0.0001					
	*X*_2_	3.09	3.09	25.35	0.0015					
	*X*_1_ *X*_2_	0.1174	0.1174	0.9613	0.3595					
	*X*_1_^2^	103.49	103.49	847.71	<0.0001					
	*X*_2_^2^	2.24	2.24	18.38	0.0036					
	Residual	0.8546	0.1221							
	Lack of Fit	0.2626	0.0875	0.5914	0.6527					
Shelf-life of ^e^ M_3_ (*Y*_3_)	Model	994.10	198.92	1065.54	<0.0001	0.9987	0.9978	0.9920	70.8166	7.97
	*X*_1_	269.30	269.30	1443.28	<0.0001					
	*X*_2_	2.44	2.44	13.06	0.0086					
	*X*_1_ *X*_2_	2.15	2.15	11.51	0.0115					
	*X*_1_^2^	112.17	112.17	601.18	<0.0001					
	*X*_2_^2^	0.4959	0.4959	2.66	0.0147					
	Residual	1.31	0.1866							
	Lack of Fit	0.7141	0.2380	1.61	0.3209					

^a^*X*_1_: packaging type; ^b^*X*_2_: OLPR, SCG, OLPR/SCG rates; ^c^ M_1_: mushrooms of WS and OLPR mixtures; ^d^ M_2_: mushrooms of WS and SCG mixtures; ^e^ M_3_: mushrooms of WS, OLPR, and SCG mixtures. Significance is considered when *p*-value < 0.05.

**Table 7:** Optimization results for extended shelf-life (days) of M_1_, M_2_, and M_3_ stored at 4 °C using the developed models.

Variable	Target	Optimum Parameter Range
*X*_1_: Packaging (type)	In-range	^a^ VBP (*Y*_1_, *Y*_2_, *Y*_3_)
*X*_2_: OLPR, SCG, OLPR/SCG (rates)	In-range	0.27 (*Y*_1_), 0.13(*Y*_2_), 0.22(*Y*_3_)
*Y*_1_: Shelf-life of ^b^ M1 (days)	Maximized	24.90
*Y*_2_: Shelf-life of ^c^ M2 (days)	Maximized	23.67
*Y*_3_: Shelf-life of ^d^ M3 (days)	Maximized	26.26

^a^ VBP: vacuum bag packaging; ^b^ M_1_: mushrooms of WS and OLPR mixtures; ^c^ M_2_: mushrooms of WS and SCG mixtures; ^d^ M_3_: mushrooms of WS, OLPR, and SCG mixtures.

**Table 8:** Total microbial count on mushrooms stored at ambient temperature (log CFU/g).

Packaging Type	Mushrooms	Total Microbial Count (Log CFU/g)										
		Day 0	Day 1	Day 2	Day 3	Day 4	Day 5	Day 6	Day 7	Day 8	Day 9	Day 10
^d^-	^a^ M_1_A	3.3	4.8	6.5	7.8	8.6	9.2	10.4	10.8	11.6	12.2	12.8
	M_1_B	2.7	4.1	5.7	7.4	7.8	8.3	8.8	9.9	11.4	12.5	13.1
	M_1_C	3.1	4.6	6.3	7.6	8.3	8.8	9.9	10.5	11.5	12.3	12.9
	^b^ M_2_A	3.3	4.8	6.5	7.8	8.6	9.2	10.3	10.9	11.8	12.7	13.4
	M_2_B	3.1	4.3	5.9	7.6	8.0	8.6	9.1	9.9	11.1	11.8	12.9
	M_2_C	3.1	4.5	6.1	7.7	8.2	8.7	9.8	10.4	11.4	12.4	13.0
	^c^ M_3_A	3.3	4.8	6.5	7.8	8.6	9.2	103	10.9	11.8	12.7	13.4
	M_3_B	2.9	4.2	5.8	7.5	7.9	8.4	8.9	10.1	11.6	12.7	13.3
	M_3_C	2.6	3.8	5.4	7.1	7.6	8.2	8.7	9.7	11.2	12.3	12.7
^e^ PBP	M_1_A	2.5	2.9	3.4	3.9	4.6	5.8	6.9	7.8	8.4	8.8	10.2
	M_1_B	2.2	2.6	3.1	3.5	4.2	5.5	6.6	7.4	8.0	8.2	8.7
	M_1_C	2.3	2.7	3.2	3.6	4.3	5.6	6.7	7.5	8.1	8.3	8.9
	M_2_A	2.7	3.1	3.6	4.1	4.8	5.7	6.9	7.5	8.0	8.5	9.1
	M_2_B	2.8	3.3	4.0	4.5	5.5	6.1	7.4	7.9	8.2	8.8	9.4
	M_2_C	2.9	3.4	4.1	4.6	5.6	6.2	7.5	8.1	8.3	8.6	9.2
	M_3_A	2.6	3.0	3.5	4.0	4.7	5.9	7.0	7.9	8.3	8.7	9.1
	M_3_B	2.1	2.7	3.3	3.9	4.2	5.1	5.9	6.7	7.9	8.2	8.6
	M_3_C	2.1	2.6	3.2	3.8	4.3	5.2	5.9	6.9	7.7	8.1	8.4
^f^ VBP	M_1_A	2.8	3.5	4.2	4.8	5.7	6.6	7.8	8.8	9.3	9.9	10.6
	M_1_B	2.3	2.9	3.2	3.8	4.4	5.0	5.8	6.6	7.8	8.0	8.3
	M_1_C	2.7	3.4	4.1	4.7	5.6	6.3	7.4	8.1	8.4	8.8	9.2
	M_2_A	3.0	3.7	4.4	5.0	5.9	6.8	8.0	8.8	9.2	9.6	10.7
	M_2_B	3.2	3.8	4.6	5.2	6.1	7.2	8.1	8.9	9.4	9.9	10.9
	M_2_C	3.4	4.2	4.9	5.7	6.5	7.7	8.8	9.4	9.9	10.6	11.4
	M_3_A	2.7	3.4	4.1	4.6	5.5	6.4	7.6	8.6	9.1	9.7	10.5
	M_3_B	2.6	3.2	3.9	4.3	5.1	6.1	7.3	7.9	8.2	8.6	8.8
	M_3_C	2.5	3.3	4.0	4.4	5.2	6.2	7.4	8.0	8.4	8.9	9.2

^a^ M_1_: mushrooms of WS and OLPR mixtures (A: WS, B: 0.3 OLPR, C: 0.7 OLPR); ^b^ M_2_: mushrooms of WS and SCG mixtures (A: WS, B: 0.3 SCG, C: 0.7 SCG); ^c^ M_3_: mushrooms of WS, OLPR, and SCG mixtures (A: WS, B: 0.17 OLPR/SCG, C: 0.33 OLPR/SCG); ^d^- no packaging; ^e^ PBP: plastic bag packaging; ^f^ VBP: vacuum bag packaging.

**Table 9:** Total microbial count on mushrooms stored at 4 °C (log CFU/g).

Packaging Type	Mushrooms	Total Microbial Count (Log CFU/g)										
		Day 0	Day 5	Day 6	Day 7	Day 15	Day 20	Day 21	Day 22	Day 23	Day 24	Day 27
^d^-	^a^ M_1_A	4.1	6.5	8.4	8.6	13.1	14.2	14.5	14.8	15.4	15.8	16.5
	M_1_B	3.6	5.6	7.3	8.1	12.3	13.5	13.9	14.4	14.9	15.7	16.2
	M_1_C	3.7	5.9	7.8	8.4	12.8	13.8	14.2	14.7	15.2	15.8	16.4
	^b^ M_2_A	4.1	6.5	8.4	8.8	13.4	14.8	15.2	15.8	16.7	17.4	17.9
	M_2_B	3.6	5.7	7.5	8.2	12.4	13.7	14.1	14.6	15.0	15.9	16.3
	M_2_C	3.8	5.9	7.9	8.5	13.0	14.0	14.3	14.6	15.3	15.5	16.0
	^c^ M_3_A	4.1	6.5	8.4	8.8	13.3	14.6	15.1	15.6	16.5	17.3	17.8
	M_3_B	3.5	5.5	7.2	8.1	11.8	12.5	13.3	13.9	14.1	14.5	15.1
	M_3_C	3.4	5.3	6.9	7.8	11.6	12.3	12.8	13.4	13.9	14.2	14.9
^e^ PBP	M_1_A	1.4	2.5	3.7	4.2	6.5	7.8	8.4	8.8	9.4	9.8	10.2
	M_1_B	1.2	1.9	2.6	3.5	5.8	7.7	7.9	8.0	8.2	8.6	9.8
	M_1_C	1.3	2.1	2.8	3.7	6.0	7.6	8.1	8.3	8.7	9.4	10.0
	M_2_A	1.4	2.5	3.7	4.2	6.5	7.8	8.4	8.9	9.4	10.2	10.9
	M_2_B	1.2	1.9	2.6	3.5	5.8	7.4	7.9	8.2	8.7	9.2	9.9
	M_2_C	1.3	2.0	2.7	3.6	5.9	7.5	8.0	8.2	8.7	9.2	9.9
	M_3_A	1.4	2.5	3.7	4.2	6.5	7.8	8.4	8.9	9.4	10.2	10.9
	M_3_B	1.1	1.7	2.4	3.3	4.9	6.2	6.7	7.3	7.9	8.2	8.6
	M_3_C	1.0	1.5	2.2	3.1	4.7	5.9	6.6	7.2	7.7	8.0	8.8
^f^ VBP	M_1_A	1.4	2.6	3.8	4.3	6.5	7.9	8.5	9.0	9.5	10.3	11.0
	M_1_B	1.1	1.6	2.3	3.2	4.7	6.0	6.5	7.1	7.7	8.2	8.7
	M_1_C	1.2	2.0	2.7	3.6	5.9	7.7	7.9	8.0	8.2	8.7	9.6
	M_2_A	1.4	2.6	3.8	4.3	6.5	7.9	8.5	9.0	9.5	10.3	10.9
	M_2_B	1.1	1.7	2.4	3.3	4.9	6.2	6.7	7.3	7.9	8.2	8.6
	M_2_C	1.2	2.0	2.7	3.6	5.9	7.7	7.9	8.0	8.2	8.7	9.4
	M_3_A	1.4	2.6	3.8	4.3	6.5	7.9	8.5	9.0	9.5	10.3	10.9
	M_3_B	1.0	1.5	2.3	3.2	4.7	5.8	6.6	7.2	7.7	8.1	8.7
	M_3_C	1.0	1.3	1.8	2.4	4.2	5.1	5.9	6.5	6.9	7.5	8.3

^a^ M_1_: mushrooms of WS and OLPR mixtures (A: WS, B: 0.3 OLPR, C: 0.7 OLPR); ^b^ M_2_: mushrooms of WS and SCG mixtures (A: WS, B: 0.3 SCG, C: 0.7 SCG); ^c^ M_3_: mushrooms of WS, OLPR, and SCG mixtures (A: WS, B: 0.17 OLPR/SCG, C: 0.33 OLPR/SCG); ^d^-: no packaging; ^e^ PBP: plastic bag packaging; ^f^ VBP: vacuum bag packaging.

## Data Availability

This article and its supplemental information files contain all the data created or analyzed during this investigation.

## Supplementary Materials

The following supporting information can be downloaded at: https://www.mdpi.com/article/10.3390/foods12030524/s1, Table S1: Physicochemical and nutritional composition variation in mushrooms stored at ambient temperature; Table S2: Physicochemical and nutritional composition variation in mushrooms stored at 4 °C.
