# Supplementary material for: Pleurotus ostreatus Grown on Agro-Industrial Residues: Studies on Microbial Contamination and Shelf-Life Prediction under Different Packaging Types and Storage Temperatures
Source: Foods. 2023 Jan 24;12(3):524. doi: 10.3390/foods12030524 (PMC9914764; doi:10.3390/foods12030524)
Supplement: Supplementary file 1 [file foods-12-00524-s001.zip › foods-2176364-supplementary.pdf]

Supplementary Data

Table S1. Physicochemical and nutritional composition variation in mushrooms stored at ambient temperature.

Packaging Type/Parameter	Mushrooms	Day 0	Day 1	Day 2	Day 3	Day 4	Day 5	Day 6	Day 7	Day 8	Day 9	Day 10
	M_1A[a]	2.0	2.8	3.7	5.0	12.5	14.5	16.5	18.5	23.5	28.5	33.5
	M_1B	1.0	1.2	2.5	3.7	4.9	5.5	12.8	14.8	16.8	21.8	26.8
	M_1C	1.4	2.1	3.2	4.9	5.4	12.6	14.6	16.6	18.6	23.6	28.6
	M_2A[b]	2.0	2.8	3.7	5.0	12.5	14.5	16.5	18.5	23.5	28.5	33.5
[d]-/WL (%)	M_2B	1.5	1.9	2.4	3.6	5.0	12.7	14.7	16.7	18.7	23.7	28.7
	M_2C	1.5	2.6	3.8	4.9	5.6	12.8	14.8	16.8	18.8	23.8	28.8
	M_3A[c]	2.0	2.8	3.7	5.0	12.5	14.5	16.5	18.5	23.5	28.5	33.5
	M_3B	1.1	1.5	2.6	3.8	4.9	5.5	12.5	14.5	16.5	21.5	26.5
	M_3C	1.0	1.4	2.5	3.7	4.8	5.1	12.1	14.1	16.1	21.1	26.1
	M_1A	0.9	1.3	1.9	2.3	2.7	3.4	4.1	4.9	5.2	11.3	17.4
	M_1B	0.8	1.1	1.4	2.0	2.4	2.8	3.5	4.2	4.9	5.6	11.7
	M_1C	0.9	1.2	1.5	2.1	2.5	2.9	3.6	4.3	4.9	5.7	11.8
	M_2A	0.9	1.3	1.9	2.3	2.7	3.4	4.1	4.9	5.2	11.3	17.4
[e]PBP/WL (%)	M_2B	0.8	1.4	2.0	2.4	2.8	3.5	4.2	4.9	5.4	11.5	16.6
	M_2C	0.8	1.6	2.2	2.6	3.0	3.7	4.4	4.9	5.6	11.7	16.8
	M_3A	0.9	1.3	1.9	2.3	2.7	3.4	4.1	4.9	5.2	11.3	17.4
	M_3B	0.7	0.8	1.1	1.7	2.1	2.5	3.2	3.9	4.6	5.3	11.4
	M_3C	0.6	0.7	1.0	1.5	1.8	2.2	2.9	3.6	4.4	4.9	6.1
	M_1A	1.5	1.9	2.2	2.7	3.2	3.7	4.2	5.0	11.5	18.0	24.5
	M_1B	0.6	0.7	1.0	1.5	1.8	2.2	2.9	3.6	4.4	4.9	6.1
	M_1C	0.8	1.6	2.2	2.6	3.0	3.7	4.4	4.9	5.6	11.7	16.8
	M_2A	1.5	1.9	2.2	2.7	3.2	3.7	4.2	5.0	11.5	18.0	24.5
[f]VBP/WL (%)	M_2B	1.6	2.3	2.8	3.3	3.8	4.3	4.9	5.6	12.1	18.6	25.1
	M_2C	1.9	2.8	3.3	3.8	4.4	5.0	11.5	18.0	24.5	31.0	37.5
	M_3A	1.5	1.9	2.2	2.7	3.2	3.7	4.2	5.0	11.5	18.0	24.5
	M_3B	0.8	1.5	2.1	2.5	2.9	3.6	4.3	4.8	5.5	11.6	16.7
	M_3C	0.9	1.2	1.5	2.1	2.5	2.9	3.6	4.3	4.9	5.7	11.8
	M_1A	7.0	6.0	4.0	3.0	2.5	2.0	1.5	1.0	1.0	1.0	1.0
	M_1B	9.0	7.0	5.0	4.5	3.5	3.0	2.5	2.0	1.5	1.0	1.0
	M_1C	8.0	7.5	5.5	3.5	3.0	2.5	2.0	1.5	1.0	1.0	1.0
	M_2A	7.0	6.0	4.0	3.0	2.5	2.0	1.5	1.0	1.0	1.0	1.0
-/Firm (N)	M_2B	8.0	7.0	5.0	4.0	3.0	2.5	2.0	1.5	1.0	1.0	1.0
	M_2C	8.0	7.5	5.5	3.5	2.5	2.0	1.5	1.0	1.0	1.0	1.0
	M_3A	7.0	6.0	4.0	3.0	2.5	2.0	1.5	1.0	1.0	1.0	1.0
	M_3B	9.0	7.0	5.0	4.5	3.5	3.0	2.5	2.0	1.5	1.0	1.0
	M_3C	9.0	8.5	7.9	5.8	3.7	2.8	2.3	1.8	1.3	1.0	1.0
	M_1A	8.0	7.4	6.3	5.2	4.7	4.2	3.7	3.2	2.9	2.4	1.9
	M_1B	9.0	8.0	6.8	5.8	5.2	4.7	4.2	3.7	3.2	2.5	2.1
	M_1C	8.0	7.5	6.7	5.6	5.0	4.5	4.0	3.5	3.1	2.4	2.1
	M_2A	8.0	7.4	6.3	5.2	4.7	4.2	3.7	3.2	2.9	2.4	1.9
PBP/Firm (N)	M_2B	8.0	7.4	6.3	5.2	4.7	4.2	3.7	3.2	2.9	2.4	1.9
	M_2C	8.0	7.3	6.2	5.1	4.6	4.1	3.6	3.1	2.8	2.3	1.8
	M_3A	8.0	7.4	6.3	5.2	4.7	4.2	3.7	3.2	2.9	2.4	1.9
	M_3B	9.0	8.1	6.9	5.9	5.3	4.8	4.3	3.8	3.3	2.6	2.2
	M_3C	9.0	8.2	7.7	7.2	6.5	6.0	5.3	4.6	3.6	3.1	2.4
VBP/Firm (N)	M_1A	8.0	7.0	5.6	4.4	4.0	3.7	3.2	2.5	2.0	1.5	1.0

	M_1B	9.0	8.2	7.7	7.2	6.5	6.0	5.3	4.6	3.6	3.1	2.4
	M_1C	8.0	7.3	6.2	5.1	4.6	4.1	3.6	3.1	2.8	2.3	1.8
	M_2A	8.0	7.0	5.6	4.4	4.0	3.7	3.2	2.5	2.0	1.5	1.0
	M_2B	9.0	7.6	5.8	4.6	4.2	3.9	3.4	2.5	2.0	1.5	1.0
	M_2C	8.5	7.2	5.5	4.0	3.5	3.0	2.5	2.0	1.5	1.0	1.0
	M_3A	8.0	7.0	5.6	4.4	4.0	3.7	3.2	2.5	2.0	1.5	1.0
	M_3B	9.0	7.4	6.3	5.2	4.7	4.2	3.7	3.2	2.8	2.3	1.8
	M_3C	9.0	7.6	6.5	5.4	4.9	4.4	3.9	3.4	3.1	2.6	2.1
	M_1A	50.0	55.0	65.0	70.0	75.0	80.0	85.0	90.0	95.0	100.0	100.0
	M_1B	40.0	50.0	55.0	60.0	65.0	75.0	80.0	85.0	90.0	95.0	100.0
	M_1C	50.0	55.0	65.0	70.0	75.0	80.0	85.0	90.0	95.0	100.0	100.0
	M_2A	50.0	55.0	65.0	70.0	75.0	80.0	85.0	90.0	95.0	100.0	100.0
-/VO (%)	M_2B	45.0	50.0	55.0	65.0	70.0	75.0	80.0	85.0	90.0	95.0	100.0
	M_2C	50.0	55.0	60.0	65.0	75.0	80.0	85.0	90.0	95.0	100.0	100.0
	M_3A	50.0	55.0	65.0	70.0	75.0	80.0	85.0	90.0	95.0	100.0	100.0
	M_3B	40.0	50.0	55.0	60.0	65.0	75.0	80.0	85.0	90.0	95.0	100.0
	M_3C	35.0	45.0	50.0	55.0	60.0	70.0	75.0	80.0	85.0	90.0	95.0
	M_1A	20.0	30.0	40.0	45.0	50.0	55.5	60.0	65.0	70.0	75.0	80.0
	M_1B	15.0	25.0	35.0	40.0	45.0	50.0	55.0	60.0	65.0	70.0	75.0
	M_1C	15.0	25.0	35.0	40.0	45.0	50.0	55.0	60.0	65.0	70.0	75.0
	M_2A	20.0	30.0	40.0	45.0	50.0	55.5	60.0	65.0	70.0	75.0	80.0
PBP/VO (%)	M_2B	20.0	30.0	40.0	45.0	50.0	55.5	60.0	65.0	70.0	75.0	80.0
	M_2C	20.0	25.0	30.0	35.0	40.0	45.0	55.0	65.0	70.0	75.0	80.0
	M_3A	20.0	30.0	40.0	45.0	50.0	55.5	60.0	65.0	70.0	75.0	80.0
	M_3B	10.0	20.0	30.0	35.0	40.0	45.0	50.0	55.0	60.0	70.0	75.0
	M_3C	0.0	10.0	20.0	30.0	35.0	40.0	45.0	50.0	60.0	65.0	70.0
	M_1A	25.0	35.0	45.0	50.0	55.0	60.0	65.0	70.0	75.0	80.0	85.0
	M_1B	0.0	10.0	20.0	30.0	35.0	40.0	45.0	50.0	60.0	65.0	70.0
	M_1C	20.0	25.0	30.0	35.0	40.0	45.0	55.0	65.0	70.0	75.0	80.0
	M_2A	25.0	35.0	45.0	50.0	55.0	60.0	65.0	70.0	75.0	80.0	85.0
VBP/VO (%)	M_2B	25.0	35.0	45.0	50.0	55.0	60.0	65.0	75.0	80.0	85.0	90.0
	M_2C	35.0	45.0	55.0	60.0	65.0	70.0	75.0	80.0	85.0	90.0	95.0
	M_3A	25.0	35.0	45.0	50.0	55.0	60.0	65.0	70.0	75.0	80.0	85.0
	M_3B	20.0	25.0	30.0	35.0	40.0	45.0	55.0	65.0	70.0	75.0	80.0
	M_3C	20.0	30.0	40.0	45.0	50.0	55.5	60.0	65.0	70.0	75.0	80.0
	M_1A	2.9	2.6	2.5	2.0	1.8	1.6	1.5	1.4	1.3	1.2	1.1
	M_1B	3.7	3.4	3.3	3.2	3.1	2.6	2.4	2.3	2.2	2.1	2.0
	M_1C	2.3	2.0	1.9	1.8	1.3	1.1	1.0	0.9	0.8	0.7	0.6
	M_2A	2.9	2.6	2.5	2.0	1.8	1.6	1.5	1.4	1.3	1.2	1.1
-/Pro (% FW)	M_2B	2.4	2.1	2.0	1.9	1.4	1.2	1.1	1.0	0.9	0.8	0.7
	M_2C	2.7	2.4	2.3	2.2	1.7	1.5	1.4	1.3	1.2	1.1	1.0
	M_3A	2.9	2.6	2.5	2.0	1.8	1.6	1.5	1.4	1.3	1.2	1.1
	M_3B	2.2	1.9	1.8	1.7	1.6	1.1	0.9	0.8	0.7	0.6	0.5
	M_3C	3.1	2.8	2.7	2.6	2.5	2.0	1.8	1.7	1.6	1.5	1.4
	M_1A	2.9	2.6	2.5	2.4	2.3	2.2	2.1	2.0	1.5	1.3	1.2
	M_1B	3.7	3.4	3.3	3.2	3.1	3.0	2.9	2.8	2.7	2.2	2.0
	M_1C	2.3	2.0	1.9	1.8	1.7	1.6	1.5	1.4	1.3	0.8	0.6
	M_2A	2.9	2.6	2.5	2.4	2.3	2.2	2.1	2.0	1.5	1.3	1.2
PBP/Pro (% FW)	M_2B	2.4	2.1	2.0	1.9	1.8	1.7	1.6	1.5	1.0	0.8	0.7
	M_2C	2.7	2.4	2.3	2.2	2.1	2.0	1.9	1.8	1.3	1.1	1.0
	M_3A	2.9	2.6	2.5	2.4	2.3	2.2	2.1	2.0	1.5	1.3	1.2
	M_3B	2.2	1.9	1.8	1.7	1.6	1.5	1.4	1.3	1.2	0.7	0.5
	M_3C	3.1	2.8	2.7	2.6	2.5	2.4	2.3	2.2	2.1	2.0	1.5
VBP/Pro (% FW)	M_1A	2.9	2.6	2.5	2.4	2.3	2.2	2.1	1.6	1.4	1.3	1.2

	M_1B	3.7	3.4	3.3	3.2	3.1	3.0	2.9	2.8	2.7	2.6	2.1
	M_1C	2.3	2.0	1.9	1.8	1.7	1.6	1.5	1.4	0.9	0.7	0.6
	M_2A	2.9	2.6	2.5	2.4	2.3	2.2	2.1	1.6	1.4	1.3	1.2
	M_2B	2.4	2.1	2.0	1.9	1.8	1.7	1.6	1.1	0.9	0.8	0.7
	M_2C	2.7	2.4	2.3	2.2	2.1	1.6	1.4	1.3	1.2	1.1	1.0
	M_3A	2.9	2.6	2.5	2.4	2.3	2.2	2.1	1.6	1.4	1.3	1.2
	M_3B	2.2	1.9	1.8	1.7	1.6	1.5	1.4	1.3	0.8	0.6	0.5
	M_3C	3.1	2.8	2.7	2.6	2.5	2.4	2.3	2.2	1.7	1.5	1.4
-/Carb (% FW)	M_1A	6.2	5.7	5.6	4.1	3.9	3.7	3.5	3.3	3.1	2.9	2.7
	M_1B	6.3	5.8	5.7	5.6	5.5	4.0	3.8	3.6	3.4	3.2	3.0
	M_1C	5.0	4.5	4.4	4.3	2.8	2.6	2.4	2.2	2.0	1.8	1.6
	M_2A	6.2	5.7	5.6	4.1	3.9	3.7	3.5	3.3	3.1	2.9	2.7
	M_2B	5.8	5.3	5.2	5.1	3.6	3.4	3.2	3.0	2.8	2.6	2.4
	M_2C	7.5	7.0	6.9	6.8	6.3	6.1	5.9	5.7	5.5	5.3	5.1
	M_3A	6.2	5.7	5.6	4.1	3.9	3.7	3.5	3.3	3.1	2.9	2.7
	M_3B	4.7	4.2	4.1	4.0	3.9	3.4	3.2	3.0	2.8	2.6	2.4
	M_3C	4.0	3.5	3.4	3.3	3.2	2.7	2.5	2.3	2.1	1.9	1.7
PBP/Carb (% FW)	M_1A	6.2	5.7	5.6	5.5	5.4	5.3	5.2	5.1	4.6	4.4	4.2
	M_1B	6.3	5.8	5.7	5.6	5.5	5.4	5.3	5.2	5.1	4.6	4.4
	M_1C	5.0	4.5	4.4	4.3	4.2	4.1	4.0	3.9	3.8	3.3	3.1
	M_2A	6.2	5.7	5.6	5.5	5.4	5.3	5.2	5.1	4.6	4.4	4.2
	M_2B	5.8	5.3	5.2	5.1	5.0	4.9	4.8	4.7	4.2	4.0	3.8
	M_2C	7.5	7.0	6.9	6.8	6.7	6.6	6.5	6.4	5.9	5.7	5.5
	M_3A	6.2	5.7	5.6	5.5	5.4	5.3	5.2	5.1	4.6	4.4	4.2
	M_3B	4.7	4.2	4.1	4.0	3.9	3.8	3.7	3.6	3.5	3.0	2.8
	M_3C	4.0	3.5	3.4	3.3	3.2	3.1	3.0	2.9	2.8	2.7	2.2
VBP/Carb (% FW)	M_1A	6.2	5.7	5.6	5.5	5.4	5.3	5.2	4.7	4.5	4.3	4.1
	M_1B	6.3	5.8	5.7	5.6	5.5	5.4	5.3	5.2	5.1	5.0	4.5
	M_1C	5.0	4.5	4.4	4.3	4.2	4.1	4.0	3.9	3.4	3.2	3.0
	M_2A	6.2	5.7	5.6	5.5	5.4	5.3	5.2	4.7	4.5	4.3	4.1
	M_2B	5.8	5.3	5.2	5.1	5.0	4.9	4.8	4.3	4.1	3.9	3.7
	M_2C	7.5	7.0	6.9	6.8	6.7	6.2	6.0	5.8	5.6	5.4	5.2
	M_3A	6.2	5.7	5.6	5.5	5.4	5.3	5.2	4.7	4.5	4.3	4.1
	M_3B	4.7	4.2	4.1	4.0	3.9	3.8	3.7	3.6	3.1	2.9	2.7
	M_3C	4.0	3.5	3.4	3.3	3.2	3.1	3.0	2.9	2.4	2.2	2.0

[a]M_1: mushrooms of WS and OLPR mixtures (A: WS, B: 0.3 OLPR, C: 0.7 OLPR); [b]M_2: mushrooms of WS and SCG mixtures (A: WS, B: 0.3 SCG, C: 0.7 SCG); [c]M_3: mushrooms of WS, OLPR, and SCG mixtures (A: WS, B: 0.17 OLPR/SCG, C: 0.33 OLPR/SCG); [d]-: no packaging; [e]PBP: plastic bag packaging; [f]VBP: vacuum bag packaging; WL: Weight Loss; Firm: Firmness; VO: Veil Opening; Pro: Protein; Carb: Carbohydrates.

Table S2. Physicochemical and nutritional composition variation in mushrooms stored at 4 °C.

Packaging Type/Parameter	Mushrooms	Day 0	Day 5	Day 6	Day 7	Day 15	Day 20	Day 21	Day 22	Day 23	Day 24	Day 27
[d]-/WL (%)	M_1A[a]	2.0	12.3	14.4	15.2	31.3	42.6	44.8	48.3	53.4	58.5	72.6
	M_1B	1.0	3.1	4.1	4.9	12.4	16.7	18.6	22.1	27.3	33.2	48.4
	M_1C	1.4	3.4	4.5	5.8	12.9	17.1	19.2	22.6	27.9	33.8	49.0
	M_2A[b]	2.0	12.3	14.4	15.2	31.3	42.6	44.8	48.3	53.4	58.5	72.6
	M_2B	1.5	3.6	4.8	6.1	13.3	17.5	19.7	23.2	28.6	34.6	49.9
	M_2C	1.5	3.7	4.7	6.0	13.3	17.6	19.8	23.3	28.7	34.7	50.2
	M_3A[c]	2.0	12.3	14.4	15.2	31.3	42.6	44.8	48.3	53.4	58.5	72.6
	M_3B	1.1	3.2	4.3	4.9	12.6	16.9	18.9	22.4	27.7	33.6	48.8
	M_3C	1.0	3.1	4.2	4.8	12.4	16.6	18.5	22.0	27.2	33.0	48.1
[e]PBP/WL (%)	M_1A	0.9	2.5	3.0	3.2	4.4	4.9	5.5	6.6	7.8	9.4	14.5
	M_1B	0.8	2.4	2.8	3.1	4.2	4.6	4.8	4.9	5.1	6.2	11.2
	M_1C	0.9	2.5	2.9	3.2	4.3	4.7	4.9	5.0	6.1	7.3	12.4
	M_2A	0.9	2.5	3.0	3.2	4.4	4.9	5.5	6.6	7.8	9.4	14.5
	M_2B	0.8	2.4	2.9	3.1	4.3	4.7	4.8	4.9	5.3	6.5	11.6
	M_2C	0.8	2.3	2.8	3.0	4.2	4.6	4.7	4.8	5.2	6.4	11.5
	M_3A	0.9	2.5	3.0	3.2	4.4	4.9	5.5	6.6	7.8	9.4	14.5
	M_3B	0.7	2.3	2.7	3.0	4.2	4.6	4.7	4.8	4.9	5.2	10.4
	M_3C	0.6	2.0	2.4	2.7	3.9	4.2	4.4	4.5	4.6	4.9	9.3
[f]VBP/WL (%)	M_1A	0.5	1.9	2.4	2.6	3.8	4.3	4.9	5.2	6.4	8.0	13.1
	M_1B	0.4	2.2	2.6	2.9	4.1	4.5	4.6	4.7	4.9	5.0	10.2
	M_1C	0.8	2.5	2.9	3.2	4.3	4.7	4.8	4.9	5.2	6.3	11.3
	M_2A	0.5	1.9	2.4	2.6	3.8	4.3	4.9	5.2	6.4	8.0	13.1
	M_2B	0.7	2.3	2.7	3.0	4.2	4.6	4.7	4.8	4.9	5.2	10.4
	M_2C	0.8	2.5	2.9	3.2	4.3	4.7	4.8	4.9	5.2	6.3	11.3
	M_3A	0.5	1.9	2.4	2.6	3.8	4.3	4.9	5.2	6.4	8.0	13.1
	M_3B	0.6	2.0	2.4	2.7	3.9	4.2	4.5	4.6	4.7	4.9	9.8
	M_3C	0.2	1.5	1.9	2.2	3.4	3.8	4.0	4.2	4.4	4.6	5.8
-/Firm (N)	M_1A	7.0	3.0	2.5	2.0	1.5	1.0	1.0	1.0	1.0	1.0	1.0
	M_1B	9.0	7.0	5.0	3.0	2.5	2.0	1.5	1.0	1.0	1.0	1.0
	M_1C	8.0	6.0	3.0	2.5	2.0	1.5	1.0	1.0	1.0	1.0	1.0
	M_2A	7.0	3.0	2.5	2.0	1.5	1.0	1.0	1.0	1.0	1.0	1.0
	M_2B	8.0	6.0	3.5	3.0	2.5	2.0	1.5	1.0	1.0	1.0	1.0
	M_2C	8.0	6.0	3.5	2.5	2.0	1.5	1.0	1.0	1.0	1.0	1.0
	M_3A	7.0	3.0	2.5	2.0	1.5	1.0	1.0	1.0	1.0	1.0	1.0
	M_3B	9.0	7.0	5.5	3.5	2.0	1.5	1.0	1.0	1.0	1.0	1.0
	M_3C	9.0	7.5	6.0	4.0	3.0	2.0	1.5	1.0	1.0	1.0	1.0
PBP/Firm (N)	M_1A	8.0	7.0	6.5	6.0	4.0	3.0	2.5	2.0	1.5	1.0	1.0
	M_1B	9.0	8.0	7.5	7.0	5.5	4.5	4.0	3.5	3.0	2.5	1.0
	M_1C	8.0	7.0	6.5	6.0	5.0	4.0	3.5	3.0	2.5	2.0	1.0
	M_2A	8.0	7.0	6.5	6.0	4.0	3.0	2.5	2.0	1.5	1.0	1.0
	M_2B	8.0	7.0	6.5	6.0	5.0	4.0	3.5	3.0	2.5	2.0	1.0
	M_2C	8.0	7.0	6.5	6.0	5.0	4.0	3.5	3.0	2.5	2.0	1.0
	M_3A	8.0	7.0	6.5	6.0	4.0	3.0	2.5	2.0	1.5	1.0	1.0
	M_3B	9.0	8.5	7.5	6.5	5.5	4.5	4.0	3.5	3.0	2.5	1.5
	M_3C	9.0	8.5	8.0	7.5	6.5	5.5	5.0	4.5	4.0	3.5	2.0
VBP/Firm (N)	M_1A	8.0	7.0	6.5	6.0	5.0	4.0	3.5	3.0	2.0	1.0	1.0
	M_1B	9.0	8.0	7.5	7.0	6.0	5.0	4.5	4.0	3.5	3.0	2.0
	M_1C	8.5	7.5	7.0	6.5	5.5	4.5	4.0	3.5	3.0	2.5	1.5

	M_2A	8.0	7.0	6.5	6.0	5.0	4.0	3.5	3.0	2.0	1.0	1.0
	M_2B	9.0	8.5	7.5	6.5	5.5	4.5	4.0	3.5	3.0	2.5	1.5
	M_2C	8.5	7.5	7.0	6.5	5.5	4.5	4.0	3.5	3.0	2.5	1.5
	M_3A	8.0	7.0	6.5	6.0	5.0	4.0	3.5	3.0	2.0	1.0	1.0
	M_3B	9.0	8.0	7.5	7.0	6.0	5.0	4.5	4.0	3.5	3.5	2.0
	M_3C	9.0	8.5	8.0	7.5	6.5	5.5	5.0	4.5	4.0	3.5	2.5
	M_1A	50.0	70.0	75.0	80.0	90.0	100.0	100.0	100.0	100.0	100.0	100.0
	M_1B	40.0	60.0	65.0	70.0	80.0	90.0	100.0	100.0	100.0	100.0	100.0
	M_1C	50.0	60.0	65.0	70.0	80.0	90.0	100.0	100.0	100.0	100.0	100.0
	M_2A	50.0	70.0	75.0	80.0	90.0	100.0	100.0	100.0	100.0	100.0	100.0
-/VO (%)	M_2B	45.0	55.0	65.0	70.0	80.0	90.0	100.0	100.0	100.0	100.0	100.0
	M_2C	50.0	60.0	65.0	70.0	80.0	90.0	100.0	100.0	100.0	100.0	100.0
	M_3A	50.0	70.0	75.0	80.0	90.0	100.0	100.0	100.0	100.0	100.0	100.0
	M_3B	35.0	55.0	60.0	70.0	80.0	90.0	100.0	100.0	100.0	100.0	100.0
	M_3C	30.0	50.0	55.0	60.0	75.0	85.0	95.0	100.0	100.0	100.0	100.0
	M_1A	20.0	40.0	45.0	50.0	60.0	70.0	75.0	80.0	85.0	90.0	100.0
	M_1B	5.0	25.0	30.0	35.0	45.0	55.0	60.0	65.0	70.0	75.0	85.0
	M_1C	10.0	30.0	35.0	40.0	50.0	60.0	65.0	70.0	75.0	80.0	90.0
	M_2A	20.0	40.0	45.0	50.0	60.0	70.0	75.0	80.0	85.0	90.0	100.0
PBP/VO (%)	M_2B	10.0	30.0	35.0	40.0	50.0	60.0	65.0	70.0	75.0	80.0	90.0
	M_2C	10.0	30.0	35.0	40.0	50.0	60.0	65.0	70.0	75.0	80.0	90.0
	M_3A	20.0	40.0	45.0	50.0	60.0	70.0	75.0	80.0	85.0	90.0	100.0
	M_3B	15.0	35.0	40.0	45.0	55.0	65.0	70.0	75.0	70.0	75.0	85.0
	M_3C	0.0	15.0	20.0	25.0	35.0	45.0	50.0	55.0	60.0	65.0	80.0
	M_1A	20.0	40.0	45.0	50.0	60.0	70.0	75.0	80.0	85.0	90.0	100.0
	M_1B	5.0	25.0	30.0	35.0	45.0	55.0	60.0	65.0	70.0	75.0	85.0
	M_1C	5.0	25.0	30.0	35.0	45.0	55.0	60.0	65.0	70.0	75.0	85.0
	M_2A	20.0	40.0	45.0	50.0	60.0	70.0	75.0	80.0	85.0	90.0	100.0
VBP/VO (%)	M_2B	15.0	35.0	40.0	45.0	55.0	65.0	70.0	75.0	70.0	75.0	85.0
	M_2C	5.0	25.0	30.0	35.0	45.0	55.0	60.0	65.0	70.0	75.0	85.0
	M_3A	20.0	40.0	45.0	50.0	60.0	70.0	75.0	80.0	85.0	90.0	100.0
	M_3B	0.0	15.0	20.0	25.0	35.0	45.0	50.0	55.0	60.0	65.0	80.0
	M_3C	0.0	10.0	15.0	20.0	30.0	40.0	45.0	50.0	55.0	60.0	75.0
	M_1A	2.9	2.4	2.3	2.2	1.5	1.0	0.9	0.8	0.7	0.6	0.3
	M_1B	3.7	3.2	3.1	3.0	2.3	1.8	1.7	1.6	1.5	1.4	1.2
	M_1C	2.3	2.0	1.9	1.8	1.3	0.9	0.8	0.7	0.6	0.5	0.2
	M_2A	2.9	2.4	2.3	2.2	1.5	1.0	0.9	0.8	0.7	0.6	0.3
-/Pro (% FW)	M_2B	2.4	2.1	2.0	1.9	1.4	1.0	0.9	0.8	0.7	0.6	0.3
	M_2C	2.7	2.4	2.3	2.2	1.7	1.3	1.2	1.1	1.0	0.9	0.6
	M_3A	2.9	2.4	2.3	2.2	1.5	1.0	0.9	0.8	0.7	0.6	0.3
	M_3B	2.2	1.7	1.6	1.5	1.0	0.7	0.6	0.5	0.4	0.3	0.1
	M_3C	3.1	2.8	2.7	2.6	2.1	1.7	1.6	1.5	1.4	1.3	1.1
	M_1A	2.9	2.7	2.6	2.5	2.0	1.8	1.7	1.6	1.5	1.4	1.2
	M_1B	3.7	3.5	3.4	3.3	2.8	2.6	2.5	2.4	2.3	2.2	2.0
	M_1C	2.3	2.1	2.0	1.9	1.4	1.2	1.1	1.0	0.9	0.8	0.6
	M_2A	2.9	2.7	2.6	2.5	2.0	1.8	1.7	1.6	1.5	1.4	1.2
PBP/Pro (% FW)	M_2B	2.4	2.2	2.1	2.0	1.5	1.3	1.2	1.1	1.0	0.9	0.7
	M_2C	2.7	2.5	2.4	2.3	1.8	1.6	1.5	1.4	1.3	1.2	1.0
	M_3A	2.9	2.7	2.6	2.5	2.0	1.8	1.7	1.6	1.5	1.4	1.2
	M_3B	2.2	2.0	1.9	1.8	1.3	1.1	1.0	0.9	0.8	0.7	0.5
	M_3C	3.1	2.9	2.8	2.7	2.2	2.0	1.9	1.8	1.7	1.6	1.4
	M_1A	2.9	2.5	2.4	2.3	1.7	1.4	1.3	1.2	1.1	1.0	0.8
VBP/Pro (% FW)	M_1B	3.7	3.3	3.2	3.1	2.5	2.2	2.1	2.0	1.9	1.8	1.6
	M_1C	2.3	2.1	2.0	1.9	1.4	1.1	1.0	0.9	0.8	0.7	0.4

	M_2A	2.9	2.5	2.4	2.3	1.7	1.4	1.3	1.2	1.1	1.0	0.8
	M_2B	2.4	2.2	2.1	2.0	1.5	1.2	1.1	1.0	0.9	0.8	0.5
	M_2C	2.7	2.5	2.4	2.3	1.8	1.5	1.4	1.3	1.2	1.1	0.9
	M_3A	2.9	2.5	2.4	2.3	1.7	1.4	1.3	1.2	1.1	1.0	0.8
	M_3B	2.2	1.9	1.8	1.7	1.2	0.9	0.8	0.7	0.6	0.5	0.3
	M_3C	3.1	2.9	2.8	2.7	2.2	1.9	1.8	1.7	1.6	1.5	1.3
-/Carb (% FW)	M_1A	6.2	5.7	5.6	5.5	4.8	4.3	4.2	4.1	4.0	3.9	3.6
	M_1B	6.3	6.0	5.9	5.8	5.1	4.6	4.5	4.4	4.3	4.2	3.9
	M_1C	5.0	4.7	4.6	4.5	3.8	3.3	3.2	3.1	3.0	2.9	2.6
	M_2A	6.2	5.7	5.6	5.5	4.8	4.3	4.2	4.1	4.0	3.9	3.6
	M_2B	5.8	5.5	5.4	5.3	4.6	4.1	4.0	3.9	3.8	3.7	3.4
	M_2C	7.5	7.2	7.1	7.0	6.3	5.8	5.7	5.6	5.5	5.4	5.2
	M_3A	6.2	5.7	5.6	5.5	4.8	4.3	4.2	4.1	4.0	3.9	3.6
	M_3B	4.7	4.4	4.3	4.2	3.5	3.0	2.9	2.8	2.7	2.6	2.3
	M_3C	4.0	3.7	3.6	3.5	2.8	2.3	2.2	2.1	2.0	1.9	1.6
PBP/Carb (% FW)	M_1A	6.2	6.0	5.9	5.8	5.3	5.1	5.0	4.9	4.8	4.7	4.5
	M_1B	6.3	6.1	6.0	5.9	5.4	5.2	5.1	5.0	4.9	4.8	4.6
	M_1C	5.0	4.8	4.7	4.6	4.1	3.9	3.8	3.7	3.6	3.5	3.3
	M_2A	6.2	6.0	5.9	5.8	5.3	5.1	5.0	4.9	4.8	4.7	4.5
	M_2B	5.8	5.6	5.5	5.4	4.9	4.7	4.6	4.5	4.4	4.3	4.1
	M_2C	7.5	7.3	7.2	7.1	6.6	6.4	6.3	6.2	6.1	6.0	5.8
	M_3A	6.2	6.0	5.9	5.8	5.3	5.1	5.0	4.9	4.8	4.7	4.5
	M_3B	4.7	4.5	4.4	4.3	3.8	3.6	3.5	3.4	3.3	3.2	3.0
	M_3C	4.0	3.8	3.7	3.6	3.1	2.9	2.8	2.7	2.6	2.5	2.3
VBP/Carb (% FW)	M_1A	6.2	5.9	5.8	5.7	5.1	4.7	4.6	4.5	4.4	4.3	4.1
	M_1B	6.3	6.1	6.0	5.9	5.3	4.9	4.8	4.7	4.6	4.5	4.3
	M_1C	5.0	4.8	4.7	4.6	4.0	3.6	3.5	3.4	3.3	3.2	3.0
	M_2A	6.2	5.9	5.8	5.7	5.1	4.7	4.6	4.5	4.4	4.3	4.1
	M_2B	5.8	5.5	5.4	5.3	4.7	4.4	4.3	4.2	4.1	4.0	3.8
	M_2C	7.5	7.2	7.1	7.0	6.4	6.3	6.2	6.1	6.0	5.9	5.7
	M_3A	6.2	5.9	5.8	5.7	5.1	4.7	4.6	4.5	4.4	4.3	4.1
	M_3B	4.7	4.4	4.3	4.2	3.6	3.2	3.1	3.0	2.9	2.8	2.6
	M_3C	4.0	3.7	3.6	3.5	2.9	2.5	2.4	2.3	2.2	2.1	1.9

[a]M_1: mushrooms of WS and OLPR mixtures (A: WS, B: 0.3 OLPR, C: 0.7 OLPR); [b]M_2: mushrooms of WS and SCG mixtures (A: WS, B: 0.3 SCG, C: 0.7 SCG); [c]M_3: mushrooms of WS, OLPR, and SCG mixtures (A: WS, B: 0.17 OLPR/SCG, C: 0.33 OLPR/SCG); [d]-: no packaging; [e]PBP: plastic bag packaging; [f]VBP: vacuum bag packaging; WL: Weight Loss; Firm: Firmness; VO: Veil Opening; Pro: Protein; Carb: Carbohydrates.
